# Aminomethylmorpholino Nucleosides as Novel Inhibitors of PARP1 and PARP2: Experimental and Molecular Modeling Analyses of Their Selectivity and Mechanism of Action

**DOI:** 10.3390/ijms252312526

**Published:** 2024-11-22

**Authors:** Irina Chernyshova, Inna Vasil’eva, Nina Moor, Nikita Ivanisenko, Mikhail Kutuzov, Tatyana Abramova, Alexandra Zakharenko, Olga Lavrik

**Affiliations:** 1 Institute of Chemical Biology and Fundamental Medicine, Siberian Branch of the Russian Academy of Sciences, 630090 Novosibirsk, Russia; chernyshova0305@gmail.com (I.C.); iva@niboch.nsc.ru (I.V.); moor@niboch.nsc.ru (N.M.); kutuzov.mm@mail.ru (M.K.); abramova@niboch.nsc.ru (T.A.); sashaz@niboch.nsc.ru (A.Z.); 2 Federal Research Centre Institute of Cytology and Genetics, Siberian Branch of the Russian Academy of Sciences, 630090 Novosibirsk, Russia; ivanisenko@bionet.nsc.ru; 3 AIRI, 123112 Moscow, Russia

**Keywords:** enzyme inhibition, PARP1, PARP2, inhibitor design, PARP trapping on DNA damage

## Abstract

Poly(ADP-ribose) polymerases 1 and 2 (PARP1 and PARP2) play a key role in DNA repair. As major sensors of DNA damage, they are activated to produce poly(ADP-ribose). PARP1/PARP2 inhibitors have emerged as effective drugs for the treatment of cancers with BRCA deficiencies. Here, we explored aminomethylmorpholino and aminomethylmorpholino glycine nucleosides as inhibitors of PARP1 and PARP2, using different enzymatic assays. The compounds bearing thymine or 5-Br(I)-uracil bases displayed the highest inhibition potency, with all of them being more selective toward PARP1. Interaction of the inhibitors with the NAD^+^ binding cavity of PARP1 (PARP2) suggested by the mixed-type inhibition was demonstrated by molecular docking and the RoseTTAFold All-Atom AI-model. The best PARP1 inhibitors characterized by the inhibition constants in the range of 12–15 µM potentiate the cytotoxicity of hydrogen peroxide by displaying strong synergism. The inhibitors revealed no impact on PARP1/PARP2 affinity for DNA, while they reduced the dissociation rate of the enzyme–DNA complex upon the autopoly(ADP-ribosyl)ation reaction, thus providing evidence that their mechanism of action for PARP trapping is due primarily to catalytic inhibition. The most active compounds were shown to retain selectivity toward PARP1, despite the reduced inhibition potency in the presence of histone PARylation factor 1 (HPF1) capable of regulating PARP1/PARP2 catalytic activity and ADP-ribosylation reaction specificity. The inhibitors obtained seem to be promising for further research as potential drugs.

## 1. Introduction

Poly(ADP-ribose) polymerases 1 and 2 (PARP1 and PARP2) are DNA-dependent nuclear regulatory NAD^+^:protein ADP-ribosyltransferases involved in an extensive network of cellular processes in higher eukaryotes. The most abundant member of PARP family enzymes, PARP1, functions as a major sensor of DNA break and plays a key role in DNA repair, transcriptional regulation, chromatin modulation, the cellular signaling pathway, inflammation, and cellular stress responses [1]. PARP1/PARP2 is activated via an allosteric switch induced by DNA binding to initiate the synthesis of poly(ADP-ribose) (PAR) using NAD^+^ as a substrate and attaching PAR both to itself (autoPARylation) and to other proteins and nucleic acids [2,3,4,5]. In human cells, PARP activity is mainly performed by PARP1 (85–90%) and also by PARP2 (10–15%) [6]. The essential functions of PARPs 1 and 2 are maintaining genome stability and cell survival overlap, since single knockouts are viable in a mouse model, in contrast to the lethal double knockout [7].

DNA repair in cancer cells reduces the efficiency of chemo- and radiotherapy. Targeting PARP1 now serves as the basis for the treatment of BRCA-deficient cancer through a mechanism named synthetic lethality [4,8,9,10,11]. It was suggested that PARP inhibitors cause an increase in DNA single-strand breaks (due to PARP trapping DNA repair intermediates), which are converted during replication to toxic DNA double-strand breaks (DSBs) irreparable in BRCA1/2 defective cells [10]. The breast cancer tumor-suppressor proteins BRCA1 and BRCA2 are important for DSB repair by homologous recombination (HR), and their dysfunction (deficiency) was shown to sensitize cells to PARP inhibition, resulting in chromosomal instability, cell cycle arrest, and subsequent apoptosis [8,9]. BRCA1 and BRCA2-defective tumors are intrinsically sensitive to PARP inhibitors, both in tumor models in vivo and in the clinic, contrasting to normal cells with an intact HR (for review, see [4,10]). The genetic interaction revealed between PARP and BRCA has been described as synthetic lethal, where the individual loss of either gene is compatible with life, but the simultaneous loss of both genes results in cell death [4,10]. Usually, the small-molecule inhibitors of PARP1 also have an inhibitory effect on PARP2 due to the high homology between the catalytic domains of these enzymes [12]. The FDA has approved five PARP inhibitors for clinical use for the treatment of breast, ovarian, prostate, and lung cancers with BRCA1/2 deficiencies: olaparib, rucaparib, niraparib, talazoparib, and veliparib [13,14]. PARP inhibitors are currently used in clinical trials to treat other diseases, either as a single inhibitor or in combination with DNA-damaging agents or immunotherapy [13,14,15,16]. However, the clinical use of PARP inhibitors known today is still limited because of their restricted bioavailability, poor physicochemical properties, insufficient efficiency in combination with traditional chemotherapy, serious toxicity and side effects, narrow application range, and drug resistance [4,17,18,19,20]. All of these shortcomings make it necessary to search for new PARP1 inhibitors and develop new drugs.

A large number of PARP inhibitors, including FDA-approved ones, has been designed based on nicotinamide to mimic the interactions between the nicotinamide ring of the NAD^+^ substrate and the enzyme active site [14]. PARP inhibitors bind to the catalytic domains of PARP1/PARP2 competing with NAD^+^, suppress the PARylation process, and prevent the release of PARP from the DNA damage site [14,20,21]. According to the available crystal structures for PARP inhibitors bound with PARP1 and PARP2 reviewed in ref. [14], three highly conserved interactions that are shared by benzamide adenine dinucleotide (BAD), olaparib, talazoparib, rucaparib, niraparib, and veliparib can be highlighted. Amino acid residues Gly863 and Ser904 in PARP1 (Gly429 and Ser470 in PARP2) are involved in the formation of hydrogen bonds with various groups of PARP inhibitors, and Tyr907 in PARP1 (Tyr473 in PARP2) is involved in π-stacking interactions with aromatic bi- or tricyclic rings of inhibitors. These interactions are important for positioning inhibitors (nicotinamide) in the active sites of the enzymes [14,22].

To create inhibitor compounds, the NAD^+^ molecule can be modified at the adenine or nicotinamide heterocyclic base, ribose moieties, or a pyrophosphate chain. According to the literature, the more selective inhibitors should bind simultaneously to the nicotinamide and adenosine binding sites of the active center of PARP enzymes [23]. Thymidine derivatives were shown in an early study to inhibit PARP1 more efficiently than 3-aminobenzamide, the structural analog of nicotinamide, which belongs to the first generation of PARP1 inhibitors [24]. Previously, we tested disaccharide nucleosides and their synthetic analogs with different carbohydrate moieties for their ability to inhibit the catalytic activity of PARP1 [25]. The lowest values of the half-maximal inhibitory concentration (IC_50_) displayed by thymidine derivatives ranged from 25 to 220 μM. Next, we developed a series of NAD^+^ mimetics represented by conjugates of ADP and nicotinamide riboside analogs in combination with an extended aliphatic linker [26,27]. The best inhibitor among them with an IC_50_ value of 42 μM contained 2-aminomethylmorpholino thymidine.

Recently, we developed new conjugates that combine morpholino nucleosides mi-micing the nicotinamide riboside fragment of the NAD^+^ molecule with ADP, and screened these compounds for potency to inhibit PARP1, PARP2, and PARP3 activities [28], since the search for inhibitors selective for each PARP remains relevant. In the two types of conjugates (phosphodiester dinucleotide pyrophosphates), morpholino nucleosides were linked to ADP through the P–O bond or the P–N bond. The derivatives with the P–N bond generally inhibited more efficiently the activity of both PARP1 and PARP2. The most active compound in the series with the P–O bond against both enzymes was the 5-I-Ura-containing analog with an IC_50_ of 255 µM for PARP1 and 160 µM for PARP2. The 5-I-Ura-containing analog in the series with the P–N bond was also among the best inhibitors with an IC_50_ of 126 µM for PARP1 and 110 µM for PARP2. But, the most active inhibitor against PARP2 from this series was the Ade-containing conjugate with an IC_50_ of 63 µM. The results demonstrate that the inhibition activity depends on the type of nitrogenous base in the conjugate, as well as on the linkage between ADP and the morpholino nucleoside. Additionally, we checked the inhibition of PARP1 and PARP2 by natural nucleosides and their derivatives [28]. Natural ribonucleosides were weakly active, and rThd was the best one with an IC_50_ value of 277 µM for PARP1 and 374 µM for PARP2. The halogen-containing derivatives inhibited both enzymes with lower IC_50_ values. The best inhibitor, 5-I-Urd, had IC_50_ values of 49 µM (PARP1) and 27 µM (PARP2). Morpholino nucleosides proved to be the more effective inhibitors of both PARP1 and PARP2 than dinucleotides and nearly the same as ribonucleosides, and the best inhibitors contained 5-iodouracil. In the present work, we explore the inhibitory potential of morpholino nucleosides against PARP1 and PARP2 enzymes. We describe the inhibition activity and the molecular modeling binding of two types of morpholino nucleoside derivatives—aminomethylmorpholino and aminomethylmorpholino glycine nucleosides—thus determining the influence of modifications in the morpholine ring on inhibition efficiency.

## 2. Results and Discussion

### 2.1. Synthesis of Aminomethylmorpholino and Aminomethylmorpholino Glycine Nucleosides

**Scheme 1:**
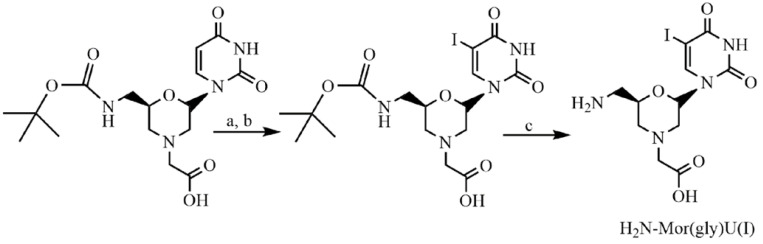
NH_2_-Mor(gly)U(I). Reaction conditions: a—ICl, MeOH, 1.5 h; b—conc. aq. NH_3_, 20 min, 0.5 M Na_2_S_2_O_3_; c—HCOOH, 2 h.

The synthesis of most of the aminomethylmorpholino and aminomethylmorpholino glycine nucleosides used in this study has been published earlier [28,29,30]. Previously, we found that the introduction of a 5′-amino group into readily available 5-I-uridine was accompanied by a significant loss of the halogen atom [31]. To prevent this, in this study we used the 2′-N-Boc-protected 2′-aminometylmorpholino glycine uracil nucleoside [29] as the parent compound for the synthesis of the 2′-aminomethylmorpholino glycine 5-I-uracyl nucleoside (Scheme 1, Figures S1 and S2).

### 2.2. Inhibition of PARP1 and PARP2 Enzymatic Activities Using Aminomethylmorpholino and Aminomethylmorpholino Glycine Nucleosides

Previously, we found disaccharide nucleosides and conjugates that combine ADP and morpholino nucleosides to inhibit PARP1/PARP2-catalyzed autopoly(ADP-ribosyl)ation (autoPARylation) reactions [25,27,28]. Based on these results, we know that the inhibitory effect depends on: (a) the introduction of the P–N bond in NAD^+^ mimetics; (b) the length and flexibility of the linker between the ADP and morpholino nucleoside; (c) the presence of a glycine fragment in the morpholino nucleoside conjugate; and d) the type of nitrogenous base (the presence of Thy or halogenated Ura was essential).

**Figure 1:**
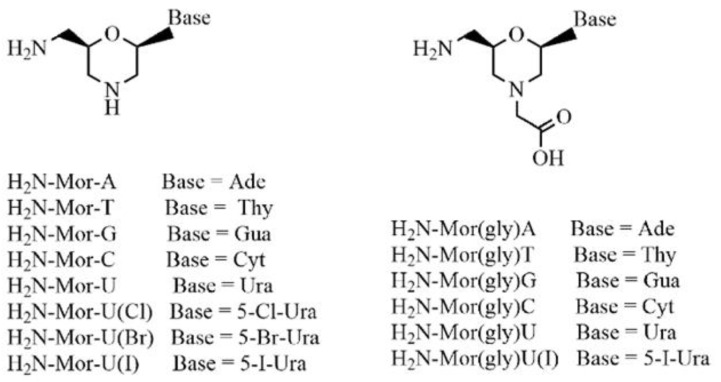
Aminomethylmorpholino and aminomethylmorpholino glycine nucleosides.

To understand the contribution of the morpholine fragment of the conjugates to the potency of PARP1/PARP2 inhibition, we explored in this study the effects of 14 aminomethylmorpholino and aminomethylmorpholino glycine mononucleosides (H_2_N-Mor-X and H_2_N-Mor(gly)X, Figure 1) on PARP1 and PARP2 activities in the autoPARylation reaction. The compounds were selected and synthesized taking into account all the listed peculiarities necessary for inhibiting PARP1/PARP2. The data of the inhibition experiments with all compounds are summarized in Table 1. The inhibition activity was revealed only for aminomethylmorpholino nucleosides with Thy and Ura bases.

The half-maximal inhibitory concentration (IC_50_) values of most inhibitors found for PARP1 and PARP2 range from 10 to 140 µM. Among the chemical characteristics desirable for a PARP inhibitor, a carboxamide group attached to an aromatic ring or embedded in an aromatic backbone, which has at least one proton on the amide nitrogen and is preferably in a cis conformation, was noted [32]. For this characteristic, thymine and uracil are the most suitable five major nucleoside bases. The glycine fragment shown previously to enhance the inhibitory efficiency of ADP-containing conjugates [27] revealed a strong negative effect on the inhibition potency of morpholino nucleoside monomers toward PARP1 (Table 1, compounds **2** and **5** vs. compounds **8** and **11**, respectively). The derivatization of the uracil moiety with a halogen atom enhanced the inhibition efficiency to a different extent, depending on the nature of the halogen atom: Cl < Br < I. A similar effect of halogenation was detected previously for the morpholino nucleoside conjugates with ADP [28].

The aminomethylmorpholino 5-Cl-uracil nucleoside (compound **12**) turned out to be a highly selective PARP1 inhibitor, despite the high homology between the PARP1 and PARP2 catalytic centers. The Br- and I-substituted counterparts also displayed a significantly higher affinity for PARP1, as evidenced by the five-eight-fold lower IC_50_ values.

Thus, the aminomethylmorpholino nucleosides turned out to be even more effective inhibitors of both PARP enzymes than the respective conjugates with ADP [28]. Apparently, this part of the previously examined conjugates is the most responsible for the inhibition against PARP1/PARP2. We were convinced once more for this series of aminomethylmorpholino nucleosides that the nature of the nitrogenous base determines the efficiency of inhibition and that Thy and 5-I(Br)-Ura are preferable components. Of all the compounds we explored here and previously for potency to inhibit PARP1 activity, compounds **8** (H_2_N-Mor-T), **13** [H_2_N-Mor-U(Br)], and **14** [H_2_N-Mor-U(I)] characterized with the lowest IC_50_ values seem to be the best inhibitors.

The four efficient inhibitors of PARP1 (compounds **8**, **12**, **13**, and **14**) were further examined by detailed kinetic experiments to determine the type of inhibition and the inhibition constants. Compound **13** revealed a strong difference in the inhibition activities against PARP1 and PARP2, which was comparatively explored in the reactions catalyzed by the two enzymes. The kinetics experiments of the PARylation reaction were performed at varied concentrations of the NAD^+^ substrate and three–four different fixed concentrations of the inhibitor. The kinetic parameters of the reaction (V_max_ and K_M_) were calculated by fitting the Michaelis–Menten equation to the kinetic data. The type of inhibition was determined by analyzing the dependences of V_max_ and K_M_ on the concentration of the inhibitor in linearizing coordinates (Figure S3). All the compounds had decreased V_max_ and increased K_M_ values as the inhibitor concentration increased (panels A−E), indicating a mixed type of inhibition, which was further evidenced by the double reciprocal plots of kinetic data (panel F). From these experiments, the inhibition constants reflecting the affinity of inhibitors to the NAD^+^ binding pocket of free enzymes were determined (Table 2).

There is no strong correlation between the K_i_ and IC_50_ values, but in the case of PARP1, they both decrease in the same order depending on the nature of the nucleoside base: U(Cl) ˃ U(Br) ˃ U(I) ≈ T. The mixed type of inhibition implies that the inhibitors can also interact with the enzyme–substrate complex and suppress enzyme activity. The respective K_i_(2) values (Table S1) are ~4−7-fold higher than the K_i_ values (Table 2), indicating a preferable interaction of the compounds tested in the NAD^+^ binding pocket.

### 2.3. Potentiation of the DNA Oxidative Agent by PARP Inhibitors

**Figure 2:**
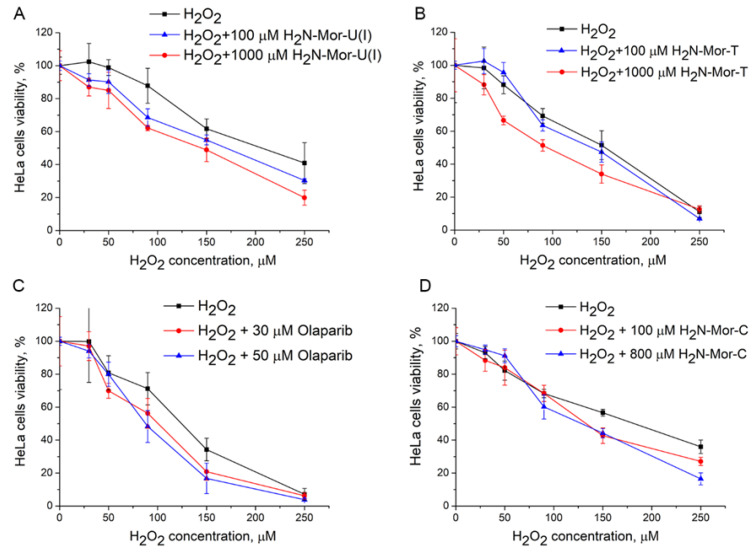
The effects of aminomethylmorpholino nucleosides and olaparib on the survival of Hela cells under conditions of oxidative stress. (**A**)–compound **14**; (**B**)–compound **8**; (**C**)–olaparib; (**D**)–compound **10**.

We studied the survival of Hela cells in different concentrations of hydrogen peroxide against the background of the most effective compounds **8** (H_2_N-Mor-T) and **14** [H_2_N-Mor-U(I)] and the approved PARP inhibitor, olaparib, using the MTT test. Cells were exposed to the oxidative agent at concentrations ranging from 30 to 250 μM against a background of 30 or 50 μM of olaparib and 100 or 1000 μM of aminomethylmorpholino nucleosides. The selected nucleosides were preliminarily proved to have no or low cytotoxicity at the range following concentrations: 100% or more than 70% cells was resistant to the treatment (Figure S4). Both olaparib and aminomethylmorpholino nucleosides enhanced the cytotoxic effects of hydrogen peroxide (Figure 2A–C, compare black graphs with blue and red ones). For all three compounds, we calculated the combination index (CI), the parameter used to determine the degree of drug interaction [33]. The CI values for the different concentrations of H_2_O_2_ and H_2_N-Mor-U(I)/H_2_N-Mor-T were less than one, and for some drug combinations less than 0.1 (Table S2). This indicates the strong synergistic effect of H_2_O_2_ and the PARP inhibitor. In the negative-control experiment with inactive compound **10** (H_2_N-Mor-C), no potentiating effect on the cytotoxicity of H_2_O_2_ was observed (Figure 2D, Table S2). Thus, only aminomethylmorpholino nucleosides acting as PARP inhibitors enhance oxidative stress caused by hydrogen peroxide in Hela cells. Notably, the CI values observed for 100 μM of H_2_N-Mor-U(I) were comparable to those for 30−50 μM of olaparib. Therefore, the difference in the concentrations of inhibitors required to elicit synergism did not reflect the much higher difference in the enzyme inhibition constants (IC_50_ and K_i_): 12−15 μM for H_2_N-Mor-U(I) and 0.4−1.3 nM for olaparib [34].

### 2.4. Impact of PARP Inhibitors on the Enzyme–DNA Interaction

Two mechanisms mediating the action of PARP1 and PARP2 inhibitors have been proposed to explain PARP trapping on the damaged DNA [35]. For the first mechanism, the inhibition of the PARP-catalyzed autoPARylation reaction impedes the dissociation of the enzyme complex with DNA. For the second mechanism, the stability of the PARP–DNA complex is allosterically enhanced by the inhibitor binding at the catalytic core independent of catalytic inhibition. To understand the mechanisms of action of our most active compounds, we explored their impact on PARP–DNA interactions in different experimental conditions.

In these studies, synthetic 5′-FAM-labeled DNA (32-mer duplex with a medially located one-nucleotide gap) was used. Its affinity for PARP1 and PARP2 was measured by fluorescence titration (Figure S5). The apparent equilibrium dissociation constants (EC_50_ values) determined by this assay for the PARP1–DNA complex in the absence and presence of H_2_N-Mor-T were almost the same (Table 3). No statistically significant effect of H_2_N-Mor-U(I) on the stability of the PARP2–DNA complex was revealed. On the contrary, olaparib had a small destabilizing effect on both PARP1 and PARP2 with DNA (1.8- and 1.6-fold increases for the respective EC_50_ values). A nearly similar effect of olaparib on the strength of PARP1-DNA binding was detected previously [34].

**Figure 3:**
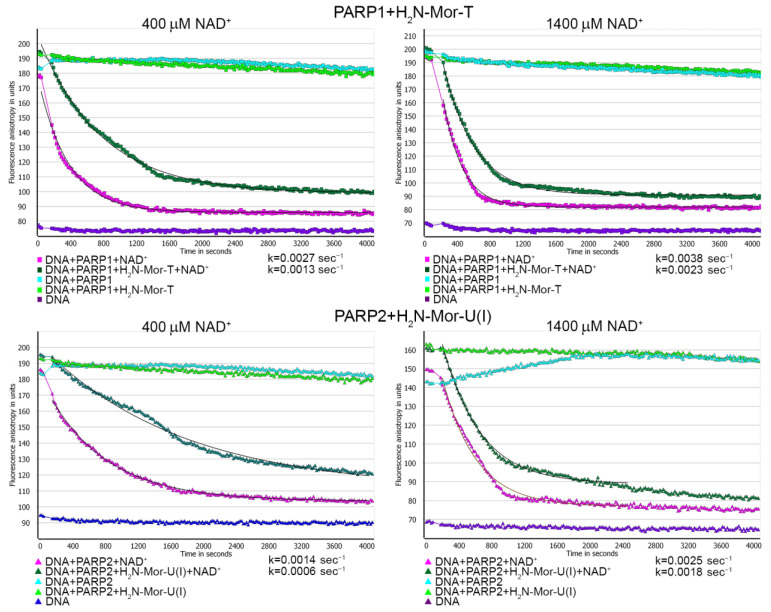
Influence of PARP inhibitors on the autoPARylation-induced dissociation of the PARP1/PARP2-DNA complex. Kinetic curves of the dissociation of PARP1 (200 nM) and PARP2 (800 nM) complexes with FAM-labeled DNA (25 nM) on the autoPARylation reaction (in the presence of 400/1400 μM NAD^+^ added at 150 s), in the absence and presence of 120 µM H_2_N-Mor-T or 240 µM H_2_N-Mor-U(I) obtained by measurements of FAM fluorescence anisotropy. The apparent dissociation rate constants for the complexes are presented in the panel legends; fitting lines to experimental data points (colored) are shown in black. The PARP1/PARP2-DNA complex is stable in the absence of NAD^+^ as shown by the respective kinetic curves (blue/light green in the absence/presence of the inhibitor, respectively).

The potential impact of inhibitors on the dissociation rate of the PARP1/PARP2 complex with DNA on the PARylation reaction was explored by the kinetic measurements of fluorescence anisotropy of FAM-DNA pre-incubated with a saturated concentration of PARP1/PARP2, in the absence or presence of the inhibitors. The addition of NAD^+^ induced the autoPARylation of PARP, which decreased the fluorescence anisotropy due to release of DNA from the complex (Figure 3). The results of these experiments show that the dissociation of the PARP1 and PARP2 complex was inhibited by aminomethylmorpholino nucleoside (H_2_N-Mor-T and H_2_N-Mor-U(I), respectively) to a different extent, depending on the NAD^+^ concentration: ~2-fold at 0.4 mM and ~1.5-fold at 1.4 mM. Analogous experiments performed for the comparison with olaparib (Figure S6) revealed more significant effects (~10-fold at 0.4 mM and ~3.5-fold at 1.4 mM), which can be explained by the high excessive inhibitor concentrations over the IC_50_ values (1/5 nM for PARP1/PARP2 [36]).

The combined results provide no evidence of an allosteric mechanism contributing to the action of our inhibitors on the PARP1/PARP2 interaction with DNA. Their trapping potency is mostly related to the inhibition of catalytic activity.

### 2.5. HPF1-Induced Modulation of the Inhibition Potency of Aminomethylmorpholino Nucleosides

Recently, Histone PARylation Factor 1 (HPF1), capable of forming a composite active site with both PARP1 and PARP2, was shown to change the PARylation reaction specificity from the modification of Glu/Asp residues to Ser residues [37,38,39]. HPF1 regulates the balance between the autoPARylation of PARP1 and PARP2 and heteroPARylation of histones.

**Figure 4:**
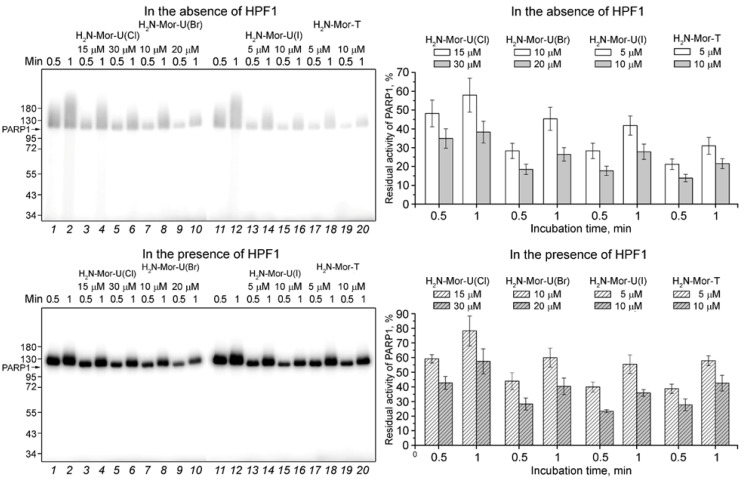
Influence of HPF1 on the potency of PARP1 inhibitors. Autoradiograms show the autoPARylation levels of PARP1 in the absence (control samples 1, 2, and 11, 12) or presence of inhibitors at the concentrations indicated, in the absence (**upper panel**) or presence of HPF1 (**lower panel**). On the left side of the image, the positions of the protein molecular weight (kDa) markers and PARP1 are indicated. Histograms show relative levels of PARP1 automodification in the presence of inhibitors at two time points of the reaction, normalized to the modification level in the respective control sample. The inhibition effects in the absence and presence of HPF1 are statistically different (*p* ˂ 0.05–0.01, calculated using a two-tailed Student’s *t*-test; *n* = 4).

PARP2 is more active during the heteroPARylation of histones than in their automodification, suggesting the specific role of PARP2 in the ADP-ribosylation-dependent modulation of the chromatin structure [40]. The most significant HPF1-induced stimulation of histone modification in the presence of gapped nucleosome-associated DNA is a peculiar feature of PARP2 [40,41]. However, the high amount of HPF1 was demonstrated to increase the amount of ADP-ribose chains synthesized by PARP1 while reducing their lengths and mediate a switch of ADP-ribosylation activity to the hydrolytic consumption of NAD^+^ [42,43]. Furthermore, it was demonstrated that HPF1 modulates the affinity of clinically relevant PARP1 inhibitors for the enzyme and PARP inhibitor cellular response [44,45,46]. This prompted us to compare the inhibition efficiency of the most active compounds in the absence and presence of HPF1. The experiments were performed on PARP1 (PARP2) and HPF1 concentrations previously observed as being optimal for the HPF1-induced stimulation of the DNA-dependent activity of PARP1 and PARP2 [40,41]. The data presented in Figure 4 show that PARP1 activity (determined as the level of enzyme automodification) is significantly enhanced due to the interaction with HPF1 in full accordance with the previously published results [40].

The inhibitors suppress the enzyme activity more efficiently (by 1.3−1.9-fold) in the absence of HPF1 than in its presence. Thus, differences in the binding modes of the inhibitors within the active site of PARP1 and the joint active site of the PARP1-HPF1 complex have no strong effect on affinity. In the case of PARP2, no impact of HPF1 on the inhibition potency of H_2_N-Mor-T, H_2_N-Mor-U(Br), and H_2_N-Mor-U(I) was detected (Figure S7). The small effect of HPF1 for PARP1 or its absence for PARP2 can be explained by the primary interaction of our inhibitors with key amino acid residues of the nicotinamide moiety binding pocket, as shown by molecular docking. The position of the nicotinamide/benzamide moiety in the crystal structures of PARP1 catalytic domain complexes with NAD^+^ analogs is independent of PARP1’s interaction with HPF1 [47]. On the other hand, the dependence of the inhibitors’ efficiency on the ADP-ribosylation specificity controlled by HPF1 seems to be a peculiar feature of PARP1.

### 2.6. Modeling of Compounds Binding to PARP1 and PARP2

**Figure 5:**
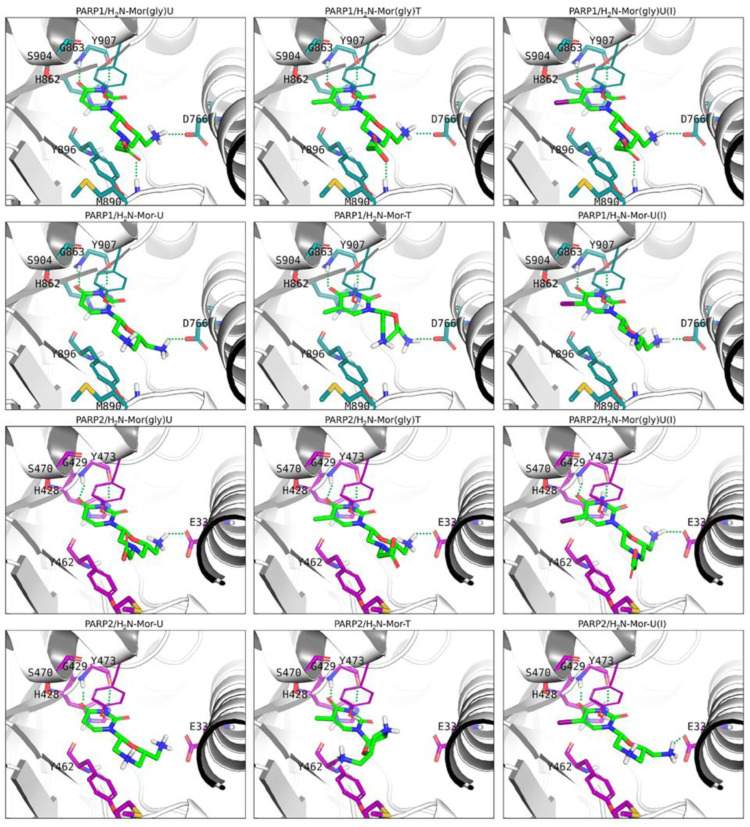
Small-molecule binding poses predicted by molecular docking using the XP score function. PARP1/PARP2 amino acid residues in contact with small molecules are shown. Small molecules are shown in green.

Molecular docking was conducted using Glide software version 5.9 and the XP scoring function [48]. The final binding poses for each ligand were chosen based on the XP score function value. The resulting molecular docking models serve as the foundation for interpreting the activity of small molecules and for the rational optimization of these compounds. According to the results, the group of compounds with a uracil moiety, H_2_N-Mor-T, H_2_N-Mor-U, H_2_N-Mor-U(Cl/Br/I), H_2_N-Mor(gly)T, H_2_N-Mor(gly)U, and H_2_N-Mor(gly)U(I), were able to form hydrogen bonds with G863/G429 residues and π-stacking interactions with Y907/Y473 residues of PARP1/PARP2, respectively (Figure 5). These pharmacophore features are known to be important for the efficient binding to the family of PARP proteins [14]. This group of compounds also demonstrated the highest SP/XP and e-model docking scores (Table S3).

Aminomethylmorpholino glycine nucleosides were able to form an extra hydrogen bond with the M890 backbone nitrogen of PARP1 compared to the aminomethylmorpholino nucleosides. In the case of aminomethylmorpholino nucleosides, such an interaction was not observed; however, one cannot exclude the presence of water-bridge-mediated binding, which was not taken into account in the molecular docking experiment. The presence of interactions with M890 in the case of PARP1 could be one of the reasons for the higher affinity toward PARP1 compared to PARP2. Additionally, both aminomethylmorpholino glycine and aminomethylmorpholino nucleosides were predicted to be able to form a salt bridge with the D766 and E335 residues of PARP1 and PARP2, respectively. The difference of small molecules in the selectivity toward PARP1 and PARP2 can also be explained by the different conformational mobility characteristics of the helical domain (HD) in addition to the amino acid composition.

According to the derived molecular models, the halogen groups of uracil derivatives and the methyl group of thymine were able to occupy the hydrophobic subpocket, which is necessary for obtaining binding free energy. This could be the reason for the higher activity of H_2_N-Mor-U(I) and H_2_N-Mor-T compared to other small molecules. Both I and CH_3_ groups have comparable van der Waals radii [49,50].

To further validate the molecular docking predictions, we employed the state-of-the-art AI-based approach: RoseTTAFold All-Atom (RFAA) [51]. This neural network facilitates the simultaneous prediction of protein structures and small-molecule binding modes. Additionally, the model offers confidence metrics, such as the predicted aligned error (PAE) between the receptor and small molecule, which estimates the accuracy of the predicted binding model. In agreement with the molecular docking predictions, RFAA validates the capability of the most active compounds to form hydrogen bonds with G863/G429 residues and π-stacking interactions with the Y907/Y473 residues of PARP1/PARP2, respectively (Figure S8). Notably, the PAE scores were generally lower for most tested small molecules in the case of PARP1 compared to PARP2. This correlation with the measured binding affinities suggests a more favorable amino acid environment for small-molecule binding in PARP1 (Table S4). Furthermore, RFAA predicted that H_2_N-Mor-U(I) and H_2_N-Mor-T would exhibit lower PAE scores compared to H_2_N-Mor(gly)U(I) and H_2_N-Mor(gly)T, aligning well with the experimental data. Overall, the confidence metrics provided by RFAA support the obtained experimental data and highlight the predictive capabilities of AI-based methods, like RoseTTAFold All-Atom. However, a drawback is that large neural networks as implemented in RFAA are prone to hallucinations [52], which, in some cases, may lead to incorrect predictions. In particular, the interaction mode of H_2_N-Mor-T with PARP2 predicted by RFAA is significantly different from the one predicted by Glide (Figure 5), or from the RFAA-predicted binding mode of the H_2_N-Mor-U(I) compound (Figure S8) characterized by an only two-fold-higher inhibition activity (Table 1). We expect that this is due to the poor performance of RFAA in this particular case.

The binding modes predicted for all the compounds tested using the two approaches are presented for comparison in Figure S9. Their analysis indicates that the primary distinction between the binding modes of active and inactive compounds lies in their capacity to mimic the natural ligand’s interactions. Active compounds can establish hydrogen bonds between the carboxamide group of the pyrimidine ring of the inhibitor and Gly863/Gly429 in the backbone chain, as well as with Ser904/Ser470 in the side chain of PARP1/PARP2, respectively. They also exhibit π-stacking interactions with Tyr907/Tyr473, enabling canonical hydrogen bonding within the active sites of PARP1 and PARP2, similar to those seen with the natural substrate NAD^+^. The absence of the carboxamide group in the adenine and cytosine rings of H_2_N-Mor(gly)A, H_2_N-Mor(gly)C, H_2_N-Mor-A, and H_2_N-Mor-C results in the significant reorientation of the nitrogenous base and/or aminomethylmorpholine ring in the respective structures and, as a consequence, in unoccupied hydrogen bonding sites in this pocket. Future studies by molecular dynamics simulations could enhance binding energy predictions and identify dynamic features essential for binding affinity improvement.

## 3. Materials and Methods

### 3.1. Chemistry

The general information, NMR, and mass spectra for the synthesis of the 2′-aminomethylmorpholino glycine 5-I-uracyl nucleoside can be found in the Supplementary Materials. H_2_N-Mor-A, H_2_N-Mor-G, H_2_N-Mor-C, H_2_N-Mor-U, H_2_N-Mor-T, H_2_N-Mor-U(Cl), H_2_N-Mor-U(I), H_2_N-Mor-U(Br), H_2_N-Mor(gly)A, H_2_N-Mor(gly)T, H_2_N-Mor(gly)G, H_2_N-Mor(gly)C, and H_2_N-Mor(gly)U were synthesized, as previously described [28,29,30,53].

Synthesis of the 2′-aminomethymorpholino glycine 5-I-uracyl nucleoside. Iodine chloride (0.06 mL, 0.5 mmol) was added to a solution of BocNH-Mor(gly)U (0.19 g, 0.5 mmol) in MeOH (2.25 mL). The mixture was stirred for 1.5 h, then conc. aq. NH_3_ (2.25 mL) was added. At 20 min, 0.5 M of Na_2_S_2_O_3_ (2.25 mL) was added. The mixture was evaporated, and EtOH (9 mL) was added to the oily residue. The precipitate formed was filtered off. The filtrate was evaporated, and the product was purified by reverse-phase chromatography (RPC) in a linear gradient of EtOH in water (0→30%). The appropriate fractions were evaporated to produce BocNH-Mor(gly)U(I). Formic acid (3 mL) was added to the residue, and the solution was evaporated at 2 h. The residue was dissolved in 20% aq. EtOH (50 mL) and the target compound was purified by cation exchange chromatography in a linear gradient of NH_4_HCO_3_ (0→0.2 M) in 20% aq. EtOH. The appropriate fractions were evaporated to produce NH_2_-Mor(gly)U(I). Yield: 0.144 g (0.35 mmol, 76%). ^1^H NMR (D_2_O): 8.19 (1H, s, H6), 5.80 (1H, dd, J 10.4, 2.4, H6′), 4.21–4.12 (1H, m, H2′), 3.33–2.92 (6H, m, H3′, H5′, NH_2_CH_2_, CH_2_-COOH), 2.45–2.37 (1H, m, H3″), and 2.25–2.16 (1H, m, H5″); ^13^C NMR (D_2_O): 178.39, 165.57, 153.30, 147.59, 81.19, 73.92, 69.99, 62.14, 55.85, 54.03, and 42.37. MS ESI (*m*/*z*): M+H^+^: calculated for C_11_H_15_IN_4_O_5_ 411.016; found: 410.600.

### 3.2. Biology

#### 3.2.1. PARP1 and PARP2 Enzyme Assays

Human recombinant poly(ADP-ribose) polymerase 1 (PARP1) and murine poly(ADP-ribose) polymerase 2 (PARP2) were expressed in insect cells and purified as previously described [54]. Radioactively labeled [^32^P]NAD^+^ was synthesized from [α-^32^P]ATP, according to the previously developed method [55]. The reaction of autopoly(ADP-ribosyl)ation was performed as follows. For PARP1, the reaction mixture contained 50 mM of Tris-HCl, a pH of 8.0, 10 mM of MgCl_2_, 150 mM of NaCl, 7 mM of β-mercaptoethanol, 2 A_260_/mL of activated DNA, 0.3 mM of [^32^P]NAD^+^, and 1 of the tested compounds. The reaction was initiated by adding PARP1 up to 200 nM, and the reaction mixtures were incubated at 37 °C for 2 min. For PARP2, the reaction mixture contained 50 mM of Tris-HCl, a pH of 8.0, 3 mM of spermine, 150 mM of NaCl, 7 mM of β-mercaptoethanol, 2 A_260_/mL of activated DNA, 0.6 mM of [^32^P]NAD^+^, and 1 of the tested compounds. The reaction was initiated by adding PARP2 up to 800 nM, and the reaction mixtures were incubated at 37 °C for 5 min. The tested compounds were added at six fixed concentrations, specified for each of them. The reaction was stopped by dropping 10 µL aliquots onto Whatman 1 filter papers soaked in 5% trichloroacetic acid (TCA). The filters were washed 4 times with 150 mL of 5% TCA and dried in the air after the removal of TCA with 90% ethanol. The introduction of radioactivity into the reaction product was quantified using a Typhoon FLA 9500 imager (GE Healthcare, Chicago, IL, USA). The half-maximal inhibitory concentration (IC_50_) values were determined using a six-point concentration response curve in three independent experiments.

#### 3.2.2. Steady-State Kinetic Analysis of PARP1 and PARP2 Enzymatic Reactions

To determine the apparent maximum rate of enzymatic reaction (V_max_), Michaelis constant (K_M_), and possible inhibition mechanism, steady-state kinetic experiments were performed at six fixed concentrations of the substrate, in the absence and presence of different fixed concentrations of the inhibitor. The standard reaction mixtures (10 μL) contained 100 μM, 300 μM, 500 μM, 1 mM, or 2 mM NAD^+^; a tested PARP1/PARP2 inhibitor specified for each compound (Figure S3); the recombinant human enzyme PARP1 (200 nM) or murine PARP2 (800 nM); and reaction buffer components (specified in the previous subsection). The reaction was performed as described in the previous subsection. The raw data (kinetic curves) were obtained in three independent experiments and statistically processed with OriginPro 8.6.0 software.

#### 3.2.3. Testing PARP1/PARP2 Inhibition in the Absence and Presence of HPF1

PARP1/PARP2 autopoly(ADP-ribosyl)ation was performed in a standard 20 μL reaction mixture containing 50 mM of Tris-HCl, a pH of 8.0, 100 mM of NaCl, 10 mM of MgCl_2_, 1 mM of DTT, 10 µM of [^32^P]NAD^+^, 1 µM of gap-DNA (a 32-mer duplex with a medially located 1-nucleotide gap), 500 nM of PARP1/PARP2 (supplemented with 1 µM of HPF1 or not), and an inhibitor of interest at concentrations specified in Figure 4 and Figure S7. The reaction was initiated by adding a mixture of [^32^P]NAD^+^ and DNA to a protein-inhibitor mixture pre-assembled on ice. After incubating the mixtures at 37 °C for the indicated time periods, the reactions were terminated by the addition of an SDS-PAGE sample buffer and heating for 2 min at 90 °C. The reaction products were analyzed using 10% SDS-PAGE with the subsequent phosphor imaging of a Coomassie brilliant blue stained gel. Protein bands labeled with [^32^P]ADP-ribose were quantified using Quantity One 4.6.7 Basic software. The residual activity (%) was determined as the level of PARP automodification in the presence of an inhibitor normalized to the respective level in the control sample containing no inhibitor.

#### 3.2.4. Fluorescence Studies of PARP1/PARP2 Interactions with DNA

Fluorescence anisotropy measurements of FAM-labeled DNA were performed in the absence and presence of various concentrations of PARP1/PARP2 with or without sub-saturating concentrations of an inhibitor. Mixtures (10 µL) containing 25 nM 5′-FAM-labeled gap-DNA (a 32-mer duplex with a medially located 1-nucleotide gap) and 20–200 nM of PARP1 or 20–800 nM of PARP2 or the enzyme mixture with the inhibitor (PARP1 with 120 µM of H_2_N-Mor-T or 400 nM of olaparib, and PARP2 with 240 µM of H_2_N-Mor-U(I) or 800 nM of olaparib) in a binding buffer (50 mM of NaCl, 50 mM of Tris-HCl, pH 8.0, and 4 of mM DTT) were prepared on ice in a Corning™ black 384-well polystyrene round-bottom low-volume plate and incubated at room temperature for 10 min. The fluorescent probes were excited at 482 nm (482–16 filter plus dichroic filter LP504), and the fluorescence intensities were detected at 530 nm (530–40 filter) using CLARIOstar spectrometer to measure the fluorescence anisotropy of FAM. Each measurement consisted of 50 flashes per well, and the resulting values of fluorescence anisotropy were automatically averaged. The averaged values were used for the final plot, and EC_50_ values were determined with MARS Data Analysis software version 3.00 (BMG LABTECH, Ortenberg, Germany). The data were plotted (F vs. C) and fitted to a four-parameter equation, F = F_0_ + (F_∞_ − F_0_)/[1 + (EC_50_/C)^n^], where F is the measured fluorescence anisotropy of a solution containing the labeled DNA at a given concentration (C) of protein, F_0_ is the fluorescence anisotropy of the solution of the labeled DNA alone, F_∞_ is the fluorescence anisotropy of the labeled DNA saturated with the protein, EC_50_ is the concentration of the protein where F − F_0_ = (F_∞_ − F_0_)/2, and n is the Hill coefficient, which denotes the slope of the nonlinear curve. The apparent rate constants (k_obs_ values) of dissociation of the PARP1 (PARP2)-DNA complex during the PARylation reaction performed in the absence or presence of an inhibitor were estimated by fluorescence anisotropy measurements, as described previously [56]. The reaction mixture (10 µL) contained 200 nM of PARP1 or 800 nM of PARP2, or the enzyme mixture with an inhibitor at a sub-saturating concentration (indicated above for each protein) and 25 nM 5′-FAM-labeled gap-DNA in the binding buffer. The reaction was initiated by the addition of 400/1400 μM of NAD^+^. The kinetic data were fitted to an exponential curve to calculate k_obs_ with MARS Data Analysis software. The fit equation is y = offset + be^kx^, where the offset is the starting anisotropy value (before NAD^+^ addition) and k is the k_obs_ value. The binding and kinetic curves show the best fit to the respective equation, with R^2^ values matching or exceeding 0.98.

#### 3.2.5. Cell Culture Cytotoxicity Assay

The cytotoxicity of the compounds and their combinations with a DNA damaging agent were examined against human cells from the HeLa cell line (cervical cancer) using an EZ4U/MTT colorimetric test (Biomedica, Vienna, Austria). The HeLa cell line was obtained from the Russian Cell Culture Collection (RCCC), Institute of Cytology RAS, St. Petersburg, Russia. The cells were grown in 96-well plates in DMEM medium (Gibco, Thermo Fisher Scientific, Waltham, MA, USA), with 1x GlutaMAX (Gibco, Thermo Fisher Scientific, Waltham, MA, USA), 50 IU/mL penicillin, 50 μg/mL streptomycin (MP Biome-dicals, Santa Ana, CA, USA), and in the presence of 10% fetal bovine serum (Biolot, Saint Petersburg, Russia) in a 5% CO_2_ atmosphere. After the formation of a 30–50% monolayer, the tested compounds were added to the medium to final concentrations specified in Figure 2 and Figure S4, and the cell culture was monitored for 3 days. The effects of the compounds on the survival of Hela cells under the condition of oxidative stress were determined in a similar way using hydrogen peroxide at concentrations of 30, 50, 90, 150, and 250 μM against the background of compounds at the indicated concentrations. The combination index (CI) values were calculated using CompuSyn software version 1.0.

### 3.3. Molecular Modeling

Molecular docking was carried out using Glide software version 5.9 (Schrödinger Drug Disco-very Suite 2017-2, Schrödinger Inc., New York, NY, USA) in the standard-precision (SP Score) and extra-precision (XP Score) modes [48]. The grid was created based on the localization of small-molecule inhibitors bound to PARP1/PARP2 in the corresponding crystal structure. Reference crystal structures of PARP1 (PDB ID 4ZZZ [57] and PARP2 (PDB ID 3KJD [58]) were used. Before docking, the 3D structures of small molecules were created using the LigPrep module and protein structures were prepared using “Protein Preparation wizard” (Schrödinger Drug Discovery Suite 2017-2, Schrödinger Inc., New York, NY, USA).

The RoseTTAFold All-Atom model was used to predict the binding poses of small molecules in the PARP1/PARP2 structure. The inference of the model was calculated using the default settings described in ref. [51], however the number of maximum iterations was set to 10. For each complex, five models were predicted, and the model with the lowest average predicted aligned error (PAE) for the intermolecular interaction of small molecules and receptors was selected.

## 4. Conclusions

The first PARP inhibitor approved as a monotherapy for the treatment of advanced ovarian cancer, olaparib, is now available for the treatment of BRCA-mutated ovarian, fallopian tube, breast, peritoneal, prostate, and metastatic pancreatic cancers. The other FDA-approved inhibitors, niraparib, rucaparib, talazoparib, and veliparib, have a narrower range of clinical applications [13,59]. A large number of PARP inhibitors of various structural classes is being developed to overcome the shortcomings of available inhibitors [4,60,61]. Novel PARP inhibitor combination therapies have been investigated to improve the efficacy of existing drugs [13,18,59,62]. The search for PARP inhibitors remains an urgent task, due to their important role in cancer therapy.

In this study, a series of novel PARP inhibitors, aminomethylmorpholino and aminomethylmorpholino glycine nucleosides, were explored. The activities of both PARP1 and PARP2 are more efficiently inhibited by aminomethylmorpholino nucleosides than by their glycine-substituted analogs and previously examined conjugates with ADP [28]. The inhibitors’ interaction with the NAD^+^ binding cavity of PARP1/PARP2 suggested by the mixed-type inhibition was demonstrated by molecular docking and the deep learning RoseTTAFold All-Atom method. The advantages of the inhibitors as potential drugs are high water solubility, the absence of cytotoxicity, and a strong synergistic effect in combination with H_2_O_2_ on Hela cell survival. The inhibitors revealed no impact on PARP1/PARP2 affinity for DNA, while they reduced the dissociation rate of the enzyme–DNA complex in the autoPARylation reaction, thus indicating that the mechanism of their action is due primarily to catalytic inhibition. Compounds **8** (H_2_N-Mor-T) and **12** [H_2_N-Mor-U(I)], which have the highest inhibition potency, are more selective toward PARP1 both in the absence and presence of HPF1 capable of modulating the enzyme active site structure and, as a consequence, catalytic activity and ADP-ribosylation specificity. In addition, the ability of HPF1 to impact the inhibition potency of our compounds was detected only for PARP1. Taken together, our results provide a new and important avenue for the future development of specific PARP1 inhibitors.

## Figures and Tables

**Table 1:** Inhibition of PARP1/PARP2 activity in the autoPARylation reaction.

Compound	PARP1 *	PARP2 *
(**1**) H_2_N-Mor(gly)A	>30%	>30%
(**2**) H_2_N-Mor(gly)T	60 ± 14	100 ± 12
(**3**) H_2_N-Mor(gly)G	>30%	>30%
(**4**) H_2_N-Mor(gly)C	>30%	>30%
(**5**) H_2_N-Mor(gly)U	>30%	>30%
(**6**) H_2_N-Mor(gly)U(I)	70 ± 12	142 ± 6
(**7**) H_2_N-Mor-A	>30%	>30%
(**8**) H_2_N-Mor-T	10 ± 3	133 ± 8
(**9**) H_2_N-Mor-G	>30%	>30%
(**10**) H_2_N-Mor-C	>30%	>30%
(**11**) H_2_N-Mor-U	420 ± 66	>30%
(**12**) H_2_N-Mor-U(Cl)	45 ± 5	>30%
(**13**) H_2_N-Mor-U(Br)	30 ± 3	250 ± 37
(**14**) H_2_N-Mor-U(I)	12 ± 2	63 ± 9

* Residual catalytic activity of PARP1 and PARP2 in the presence of a 1 mM compound (%) or the IC_50_ (μM) is presented. Values are the means (±SDs) of three independent experiments.

**Table 2:** The inhibition constants of the most active compounds against PARP1/PARP2.

Compound	K_i_ *, μM	Enzyme
(**8**) H_2_N-Mor-T	12 ± 2	PARP1
(**12**) H_2_N-Mor-U(Cl)	79 ± 6	PARP1
(**13**) H_2_N-Mor-U(Br)	25 ± 4	PARP1
(**14**) H_2_N-Mor-U(I)	15 ± 3	PARP1
(**14**) H_2_N-Mor-U(I)	120 ± 23	PARP2

* All tested compounds appear to be mixed-type inhibitors. The K_i_ value described in the Supplementary Materials reflects an affinity for the free enzyme.

**Table 3:** Apparent equilibrium dissociation constants (EC_50_ values) of PARP complexes with DNA in the absence and presence of inhibitors.

Compound	Enzyme	EC_50_ *, nM
-	PARP1	36 ± 4
olaparib	PARP1	63 ± 7
(**8**) H_2_N-Mor-T	PARP1	40 ± 5
-	PARP2	150 ± 10
olaparib	PARP2	250 ± 36
(**14**) H_2_N-Mor-U(I)	PARP2	170 ± 22

* The presented data are the means (±SDs) of 2 independent experiments run in duplicate.

## Data Availability

The original contributions presented in the study are included in the article/Supplementary Materials, further inquiries can be directed to the corresponding author.

## Supplementary Materials

The following supporting information can be downloaded at: https://www.mdpi.com/article/10.3390/ijms252312526/s1.

## Abbreviations

PARP	Poly(ADP-ribose) polymerase
PARylation	Poly(ADP-ribosyl)ation
FAM	Fluorescein
HPF1	Histone PARylation Factor 1
RFAA	RoseTTAFold All-Atom
