# Supplementary material for: Aminomethylmorpholino Nucleosides as Novel Inhibitors of PARP1 and PARP2: Experimental and Molecular Modeling Analyses of Their Selectivity and Mechanism of Action
Source: Int J Mol Sci. 2024 Nov 22;25(23):12526. doi: 10.3390/ijms252312526 (PMC11640836; doi:10.3390/ijms252312526)
Supplement: Supplementary file 1 [file ijms-25-12526-s001.zip › ijms-3293057-supplementary.pdf]

Supplementary Materials

Aminomethylmorpholino Nucleosides as Novel Inhibitors of PARP1 and PARP2: Experimental and Molecular Modeling Analyses of their Selectivity and Mechanism of Action

Irina Chernyshova [1,†], Inna Vasil'eva [1], Nina Moor [1,†], Nikita Ivanisenko [2,3], Mikhail Kutuzov [1], Tatyana Abramova [1], Alexandra Zakharenko [1] and Olga Lavrik [1,*]

[1] Institute of Chemical Biology and Fundamental Medicine, Siberian Branch of the Russian Academy of Sciences, 630090 Novosibirsk, Russia; chernyshova0305@gmail.com (I.C.); iva@niboch.nsc.ru (I.V.); moor@niboch.nsc.ru (N.M.); kutuzov.mm@mail.ru (M.K.); abramova@niboch.nsc.ru (T.A.); sashaz@niboch.nsc.ru (A.Z.)
[2] Federal Research Centre Institute of Cytology and Genetics, Siberian Branch of the Russian Academy of Sciences, 630090 Novosibirsk, Russia; ivanisenko@bionet.nsc.ru
[3] AIRI, 123112 Moscow, Russia
***** Correspondence: lavrik@niboch.nsc.ru
† These authors contributed equally to the paper

General, chemistry

Reagents and solvents were purchased from Sigma-Aldrich (USA), Alfa Aesar (USA) and Reachem (Russia). Organic solvents were dried and purified by standard procedures. NMR spectra were acquired on Bruker AM-400 instruments (Bruker, Germany) in D_2O at 30 °C. Chemical shifts (δ) are reported in ppm relative to the TMS signal. Coupling constants *J* are reported in Hertz. ESI mass spectra were registered on Agilent ESI MSD XCT Ion Trap (Agilent Technologies, USA) in positive mode at The Center of Cooperative Use ("Proteomics", Russian Academy of Sciences). Monitoring of a reaction progress and quantitative analytical HPLC were performed on a Milichrom A02 chromatograph system equipped with the MultiChrom program package (Econova, Russia) on a ProntoSIL 125 C18 column (2 × 75 mm) in a gradient of buffer B (0.1 M TEA–AcOH, pH 7.0, 80% MeCN) in buffer A (0.1 M TEA–AcOH, pH 7.0, water) with an elution rate of 0.2 mL/min and UV detection at 250, 260, 280, and 300 nm. TLC was carried out on Kieselgel 60 F254 plates (Merck, Germany) in the proper solvent systems and visualized by UV irradiation, ninhydrin (amine groups). RPC and cation exchange chromatography were performed using Poligoprep 100-50 C18 (Macherey-Nagel, Germany) and Servacel P-23 (Serva, USA), respectively. The compositions of all liquid mixtures are indicated as (v/v) percent. All evaporations were performed under reduced pressure.

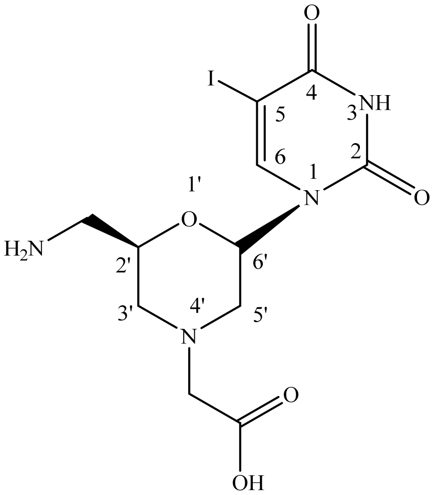

Figure S1. Structure of 2'-aminomethymorpholino glycine 5-I-uracyl nucleoside.

^{1}H NMR

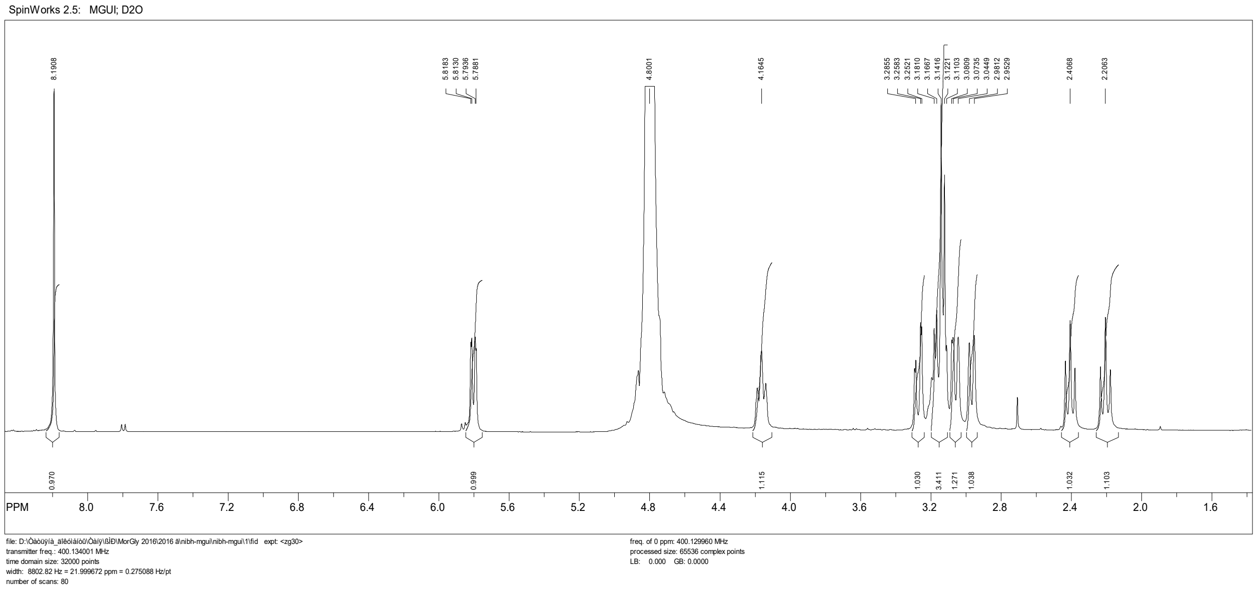

^{13}C NMR

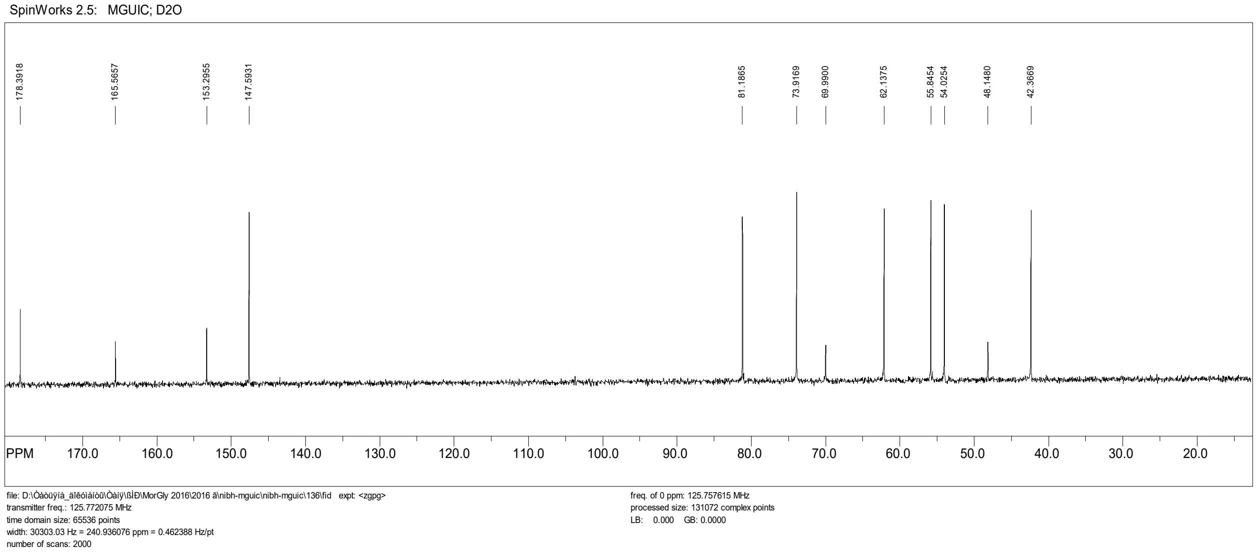

Mass-spectrum

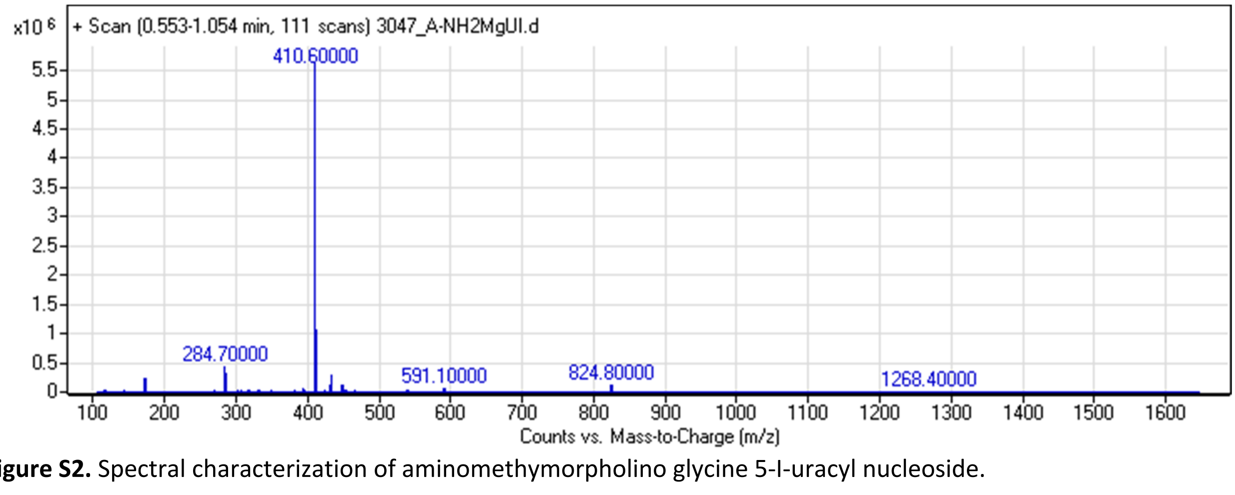

Figure S2. Spectral characterization of aminomethymorpholino glycine 5-I-uracyl nucleoside.

A) PARP1 inhibition by H_2N-Mor-T. V_{max} ↓, K_M ↑ - mixed-type inhibition

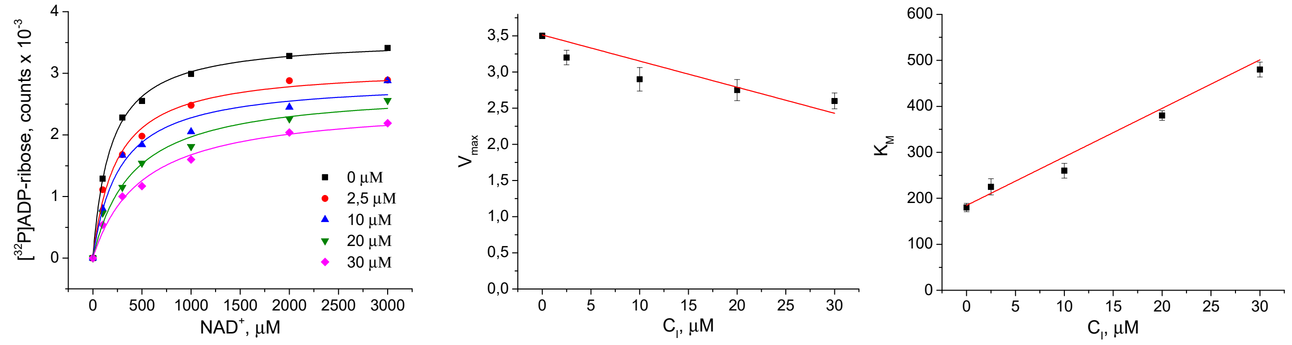

B) PARP1 inhibition by H_2N-Mor-U(Cl). V_{max} ↓, K_M ↑ - mixed-type inhibition.

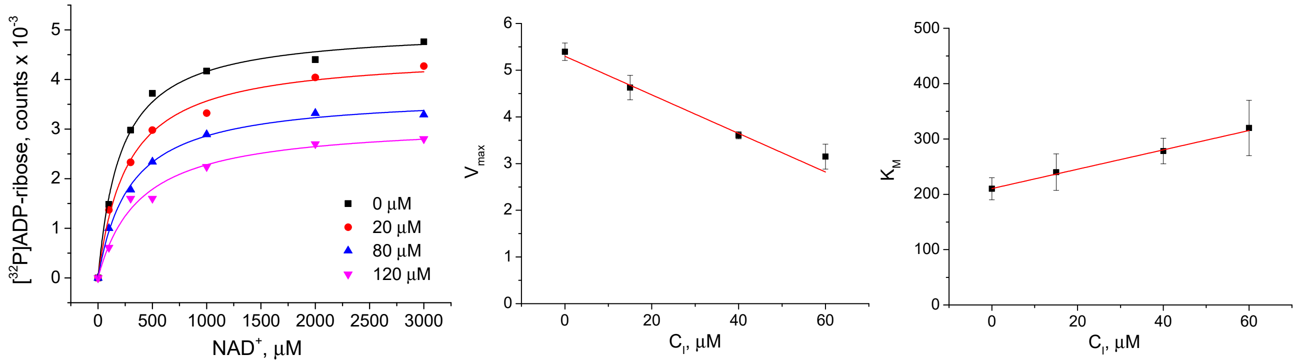

C) PARP1 inhibition by H_2N-Mor-U(Br). V_{max} ↓, K_M ↑ - mixed-type inhibition.

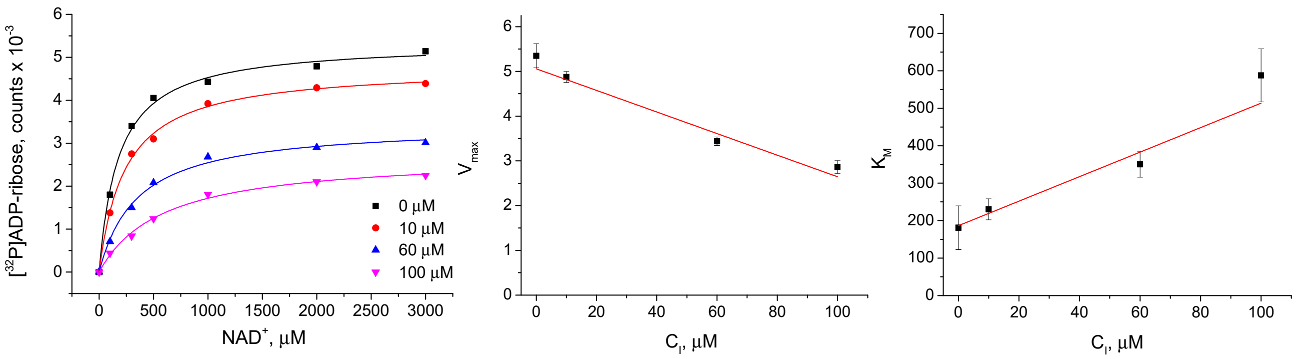

D) PARP1 inhibition by H_2N-Mor-U(I). V_{max} ↓, K_M ↑ - mixed-type inhibition.

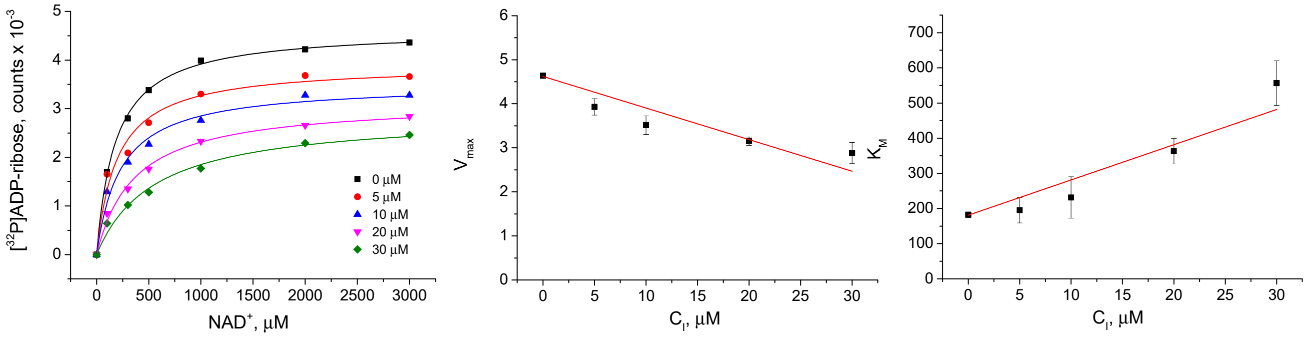

E) PARP2 inhibition by H_2N-Mor-U(I). V_{max} ↓, K_M ↑ - mixed-type inhibition.

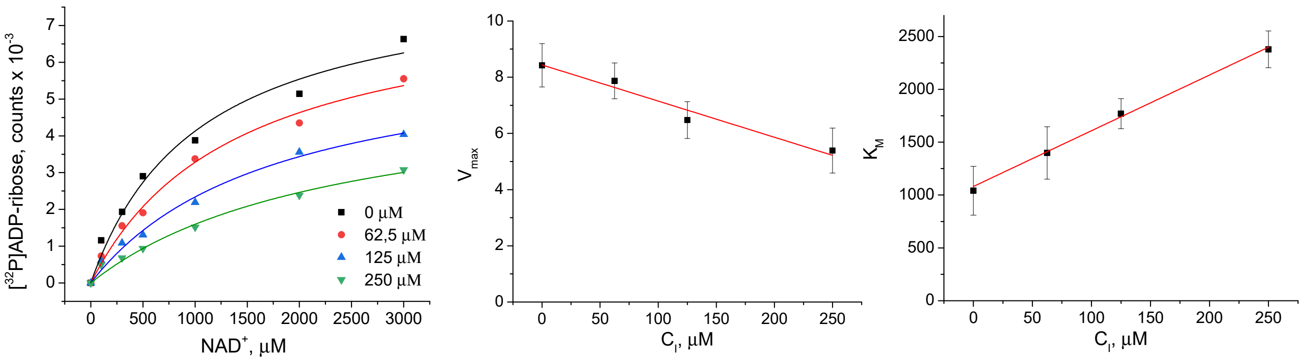

F) Graphical representation in double reciprocal plots. The inhibitor concentrations (µM) are indicated right to each panel.

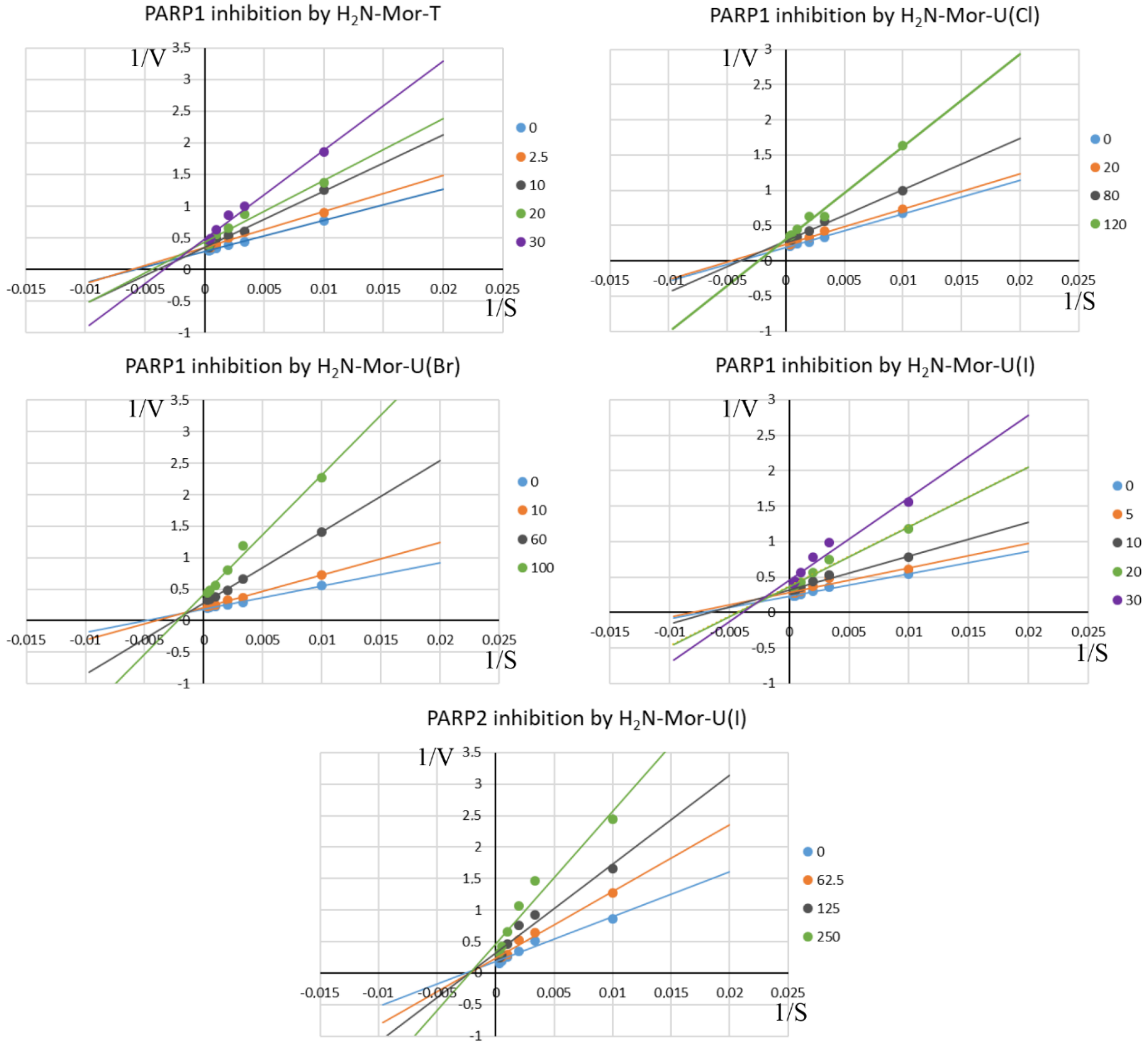

Figure S3. Graphical representation of inhibition type determination for PARP1 and PARP2 with selected compounds. The Ki and Ki(2) values presented in Table 2 in the main text and Table S1 were calculated using the equations:

$$Ki = \frac{Ci}{\left[{}^{(Vmax/Km)}\Big/_{(Vmax*/Km*)}\right] - 1} \qquad Ki(2) = \frac{Ci}{{}^{V}\!/_{Vmax*} - 1}$$

where *Vmax* (*Vmax**) – maximal reaction velocity in the absence (presence) of inhibitor; *Km* (*Km**) – Michaelis constant in the absence (presence) of inhibitor; *Ci* – the inhibitor concentration.

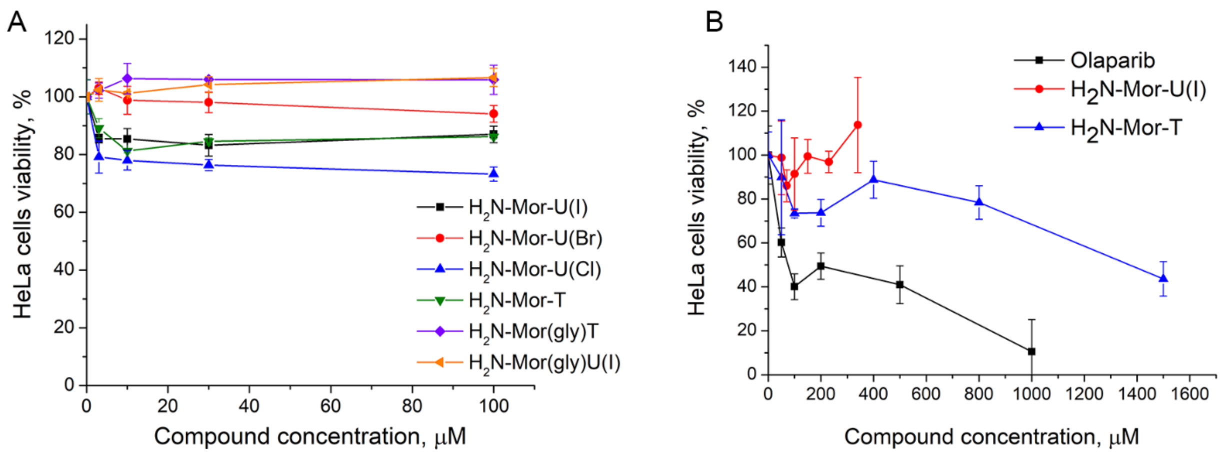

Figure S4. Cytotoxicity of PARP1/PARP2 inhibitors towards Hela cells. The cell viability was measured with the EZ4U (A) or MTT (B) colorimetric test assay.

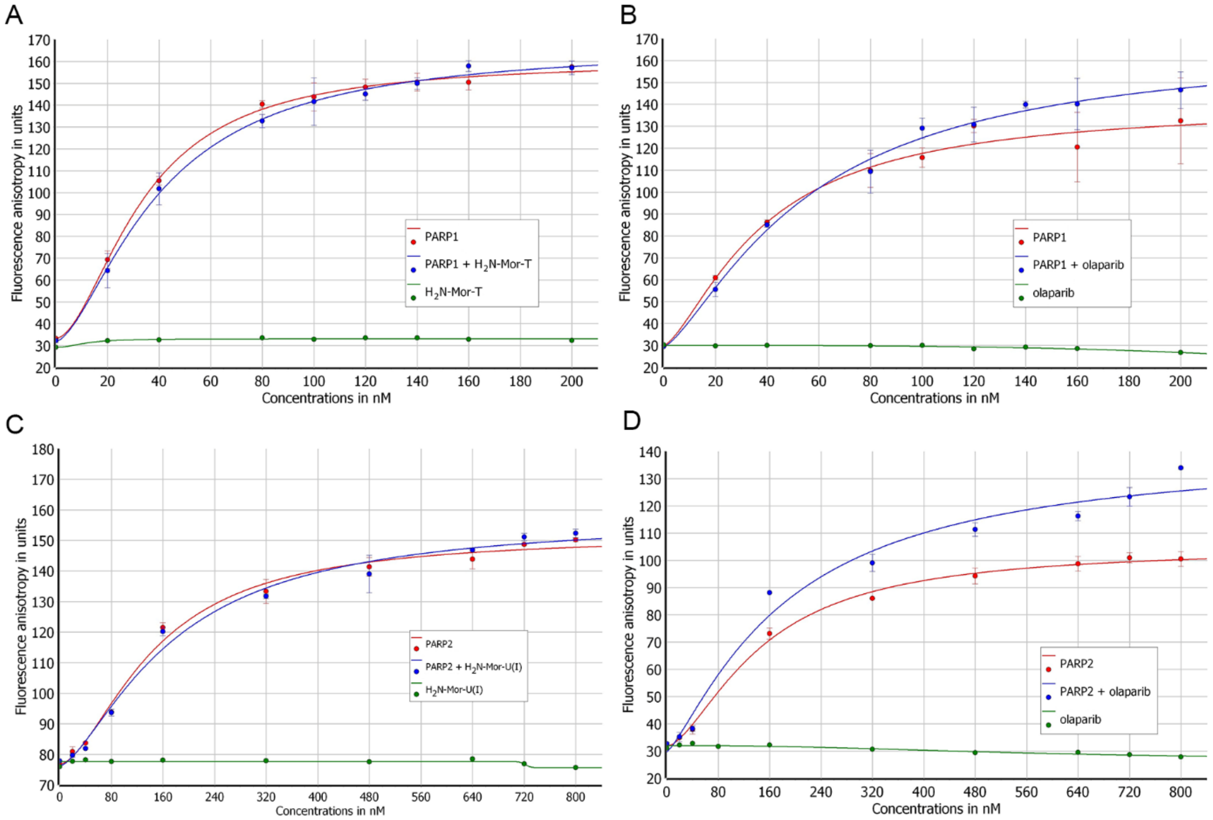

Figure S5. Modulation of PARP1/PARP2 affinity for DNA by inhibitors. Typical titration curves obtained by fluorescence anisotropy measurements represent the binding of FAM-labeled DNA (25 nM) to PARP1 (A, B) and PARP2 (C, D) at varied concentrations of the enzymes, in the absence or presence of inhibitors specified in the panels. The inhibitors were added to the enzymes at sub-saturating concentrations: 120 µM H_2N-Mor-T (A), 240 µM H_2N-Mor-U(I) (C), 400/800 nM olaparib (B/D).

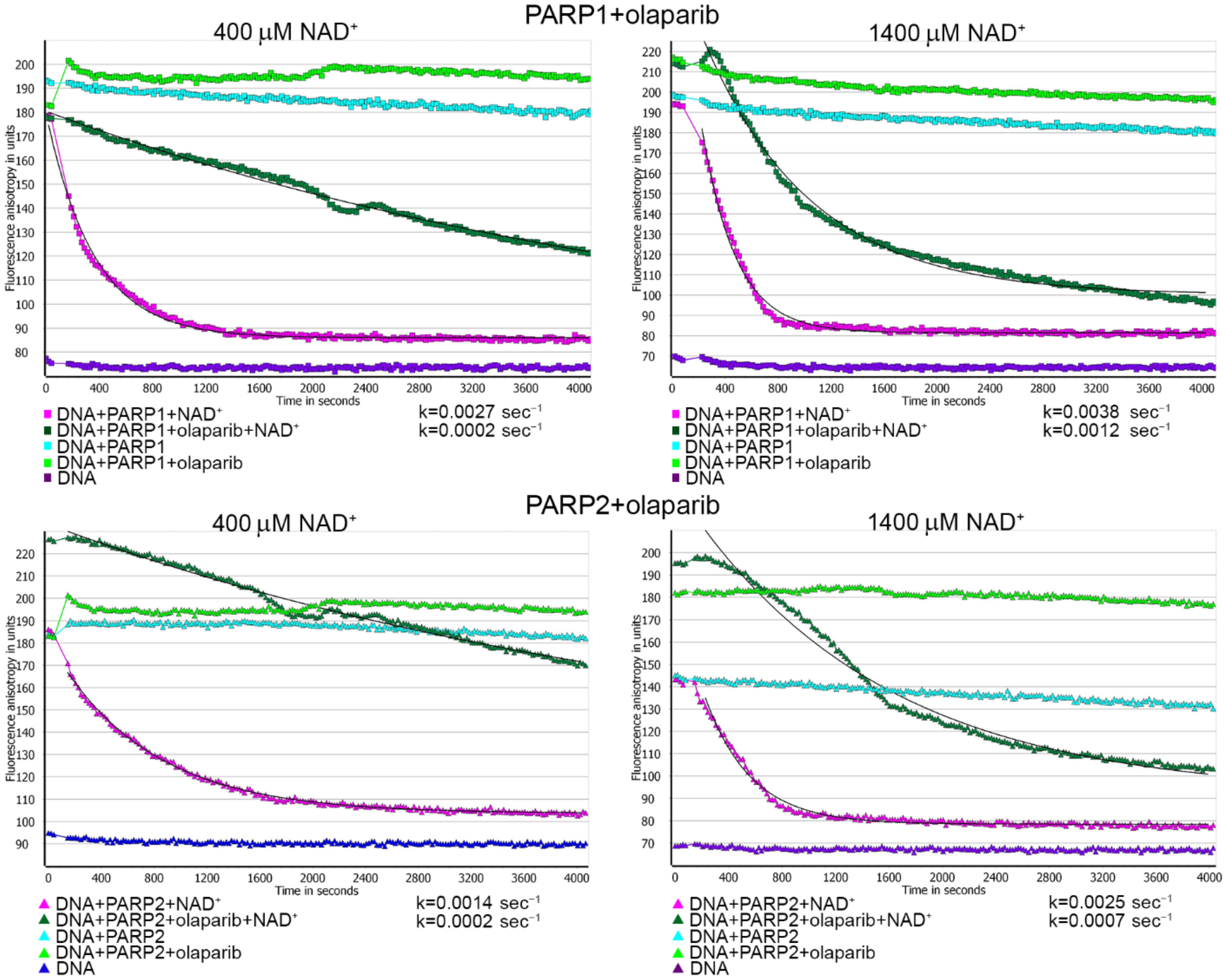

Figure S6. Influence of olaparib on autoPARylation-induced dissociation of PARP1/PARP2-DNA complex. Kinetic curves of dissociation of PARP1 (200 nM) and PARP2 (800 nM) complexes with FAM-labeled DNA (25 nM) upon the autoPARylation reaction (in the presence of 400/1400 μM NAD^+ added at 150 s), in the absence and presence of 400/800 nM olaparib (for PARP1/PARP2) obtained by measurements of FAM fluorescence anisotropy. The apparent dissociation rate constants for the complexes are presented in the panel legends; fitting lines to experimental data points (colored) are shown in black. PARP1/PARP2-DNA complex is stable in the absence of NAD^+ as shown by the respective kinetic curves (blue/light green in the absence/presence of inhibitor).

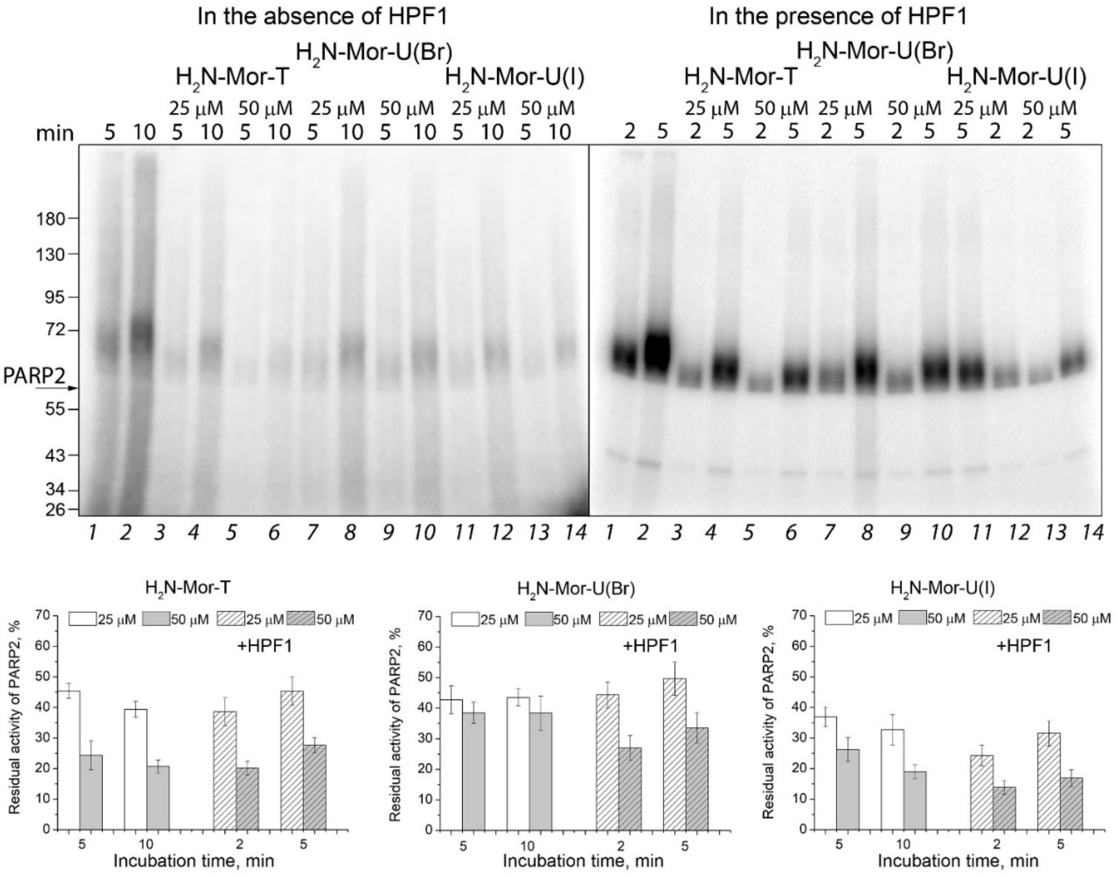

Figure S7. Influence of HPF1 on the potency of PARP2 inhibitors. Autoradiograms show autoPARylation levels of PARP2 in the absence (control samples 1, 2) or presence of inhibitors at the concentrations indicated. On the left side of the gel image positions of protein molecular weight (kDa) markers and PARP2 are indicated. Histograms show relative levels of PARP2 automodification in the presence of inhibitors at two time points of the reaction and two concentrations of each inhibitor, normalized to the modification level in the respective control sample. The differences in the residual enzyme activity in the absence and presence of each inhibitor (determined at identical incubation time, i.e. 5 min) were statistically insignificant (calculated by a two-tailed Student's t-test; n=3).

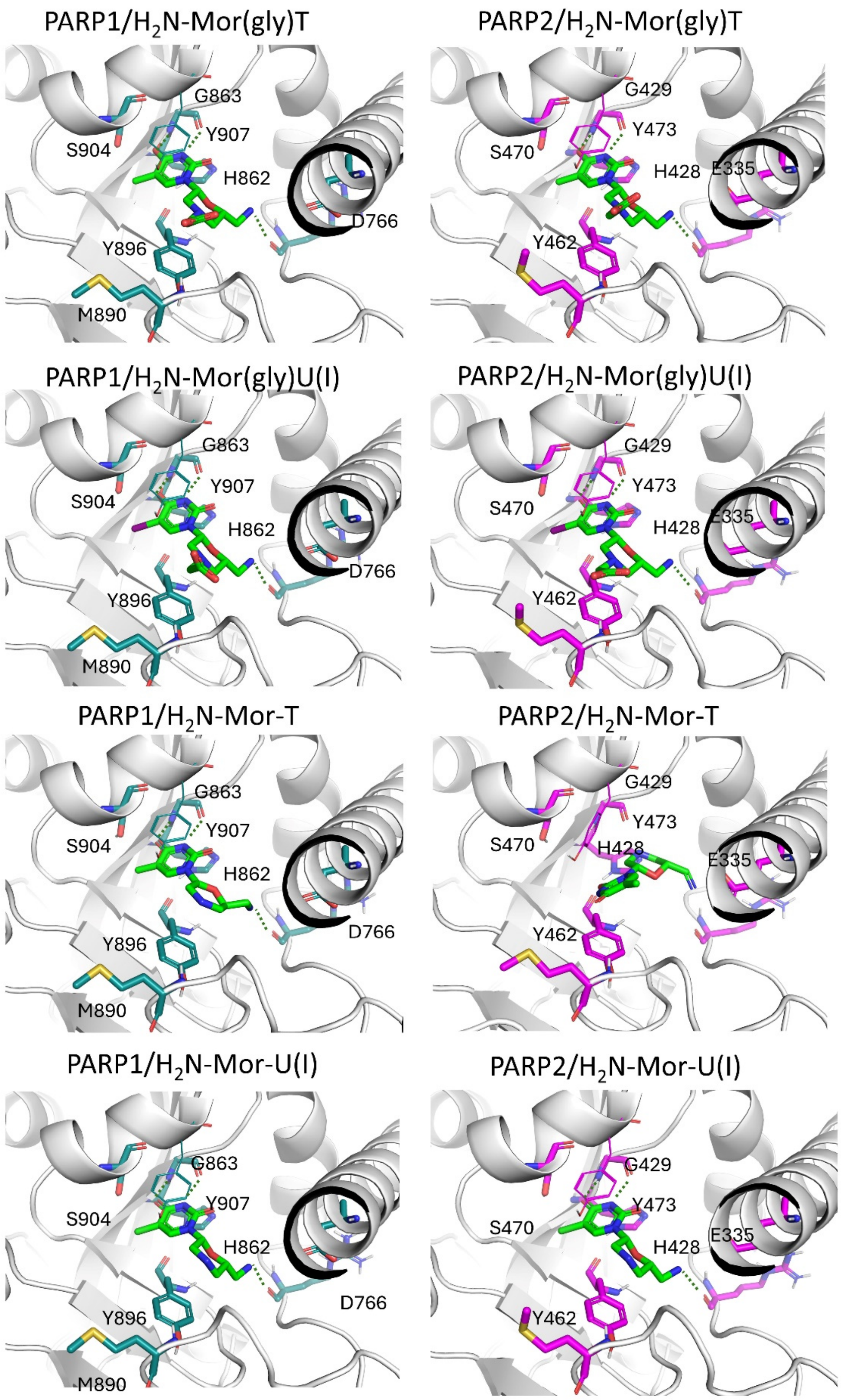

Figure S8. Small molecule binding poses predicted by RoseTTAFold All-Atom. PARP1/PARP2 amino acid residues in contact with small molecules are denoted. Small molecules are shown in green.

A) Predicted binding modes of compounds using Glide with the XP scoring function:

PARP1/H_2N-Mor(gly)T

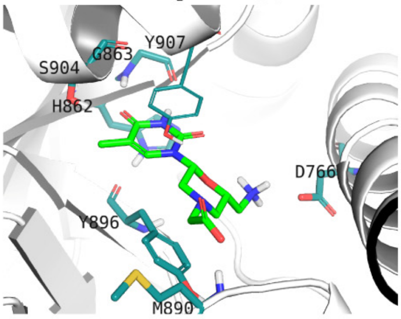

PARP1/H_2N-Mor(gly)U(I)

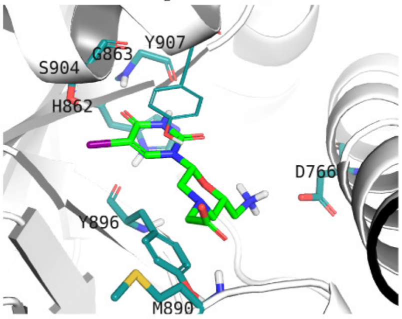

PARP1/H_2N-Mor(gly)U

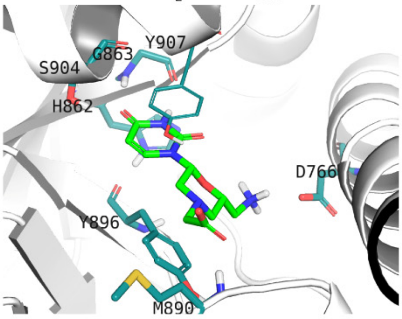

PARP1/H_2N-Mor(gly)C

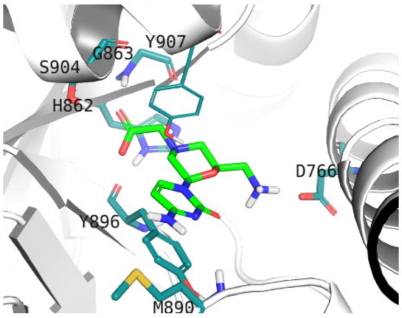

PARP1/H_2N-Mor(gly)A

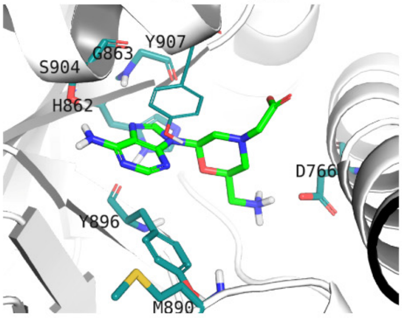

PARP1/H_2N-Mor(gly)G

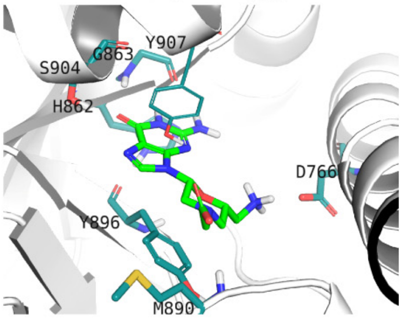

PARP1/H_2N-Mor-T

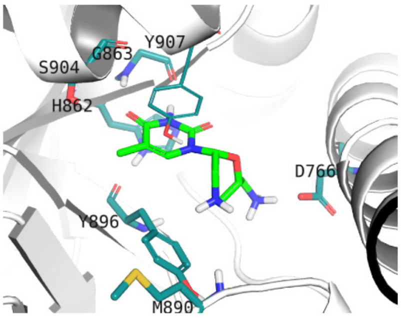

PARP1/H_2N-Mor-U(I)

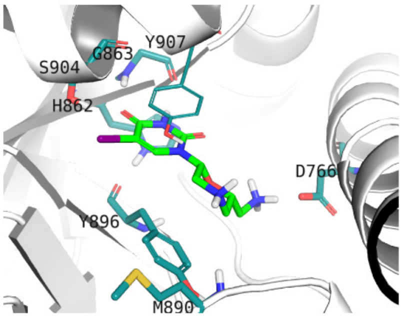

PARP1/H_2N-Mor-U(Br)

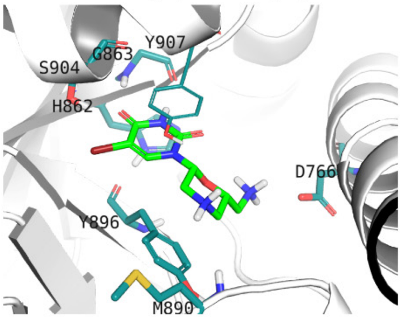

PARP1/H_2N-Mor-U(Cl)

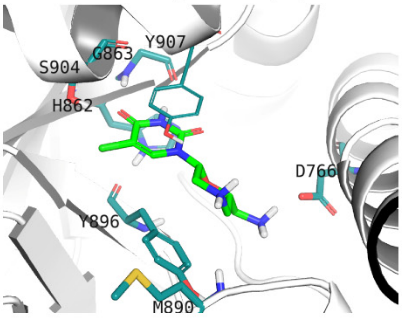

PARP1/H_2N-Mor-U

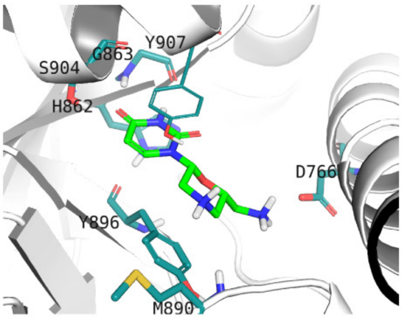

PARP1/H_2N-Mor-C

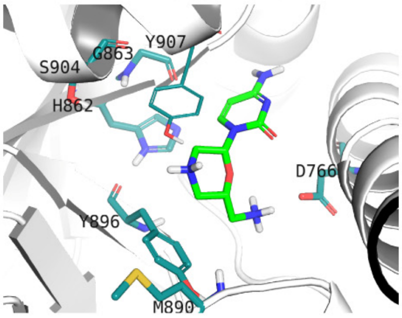

PARP1/H_2N-Mor-A

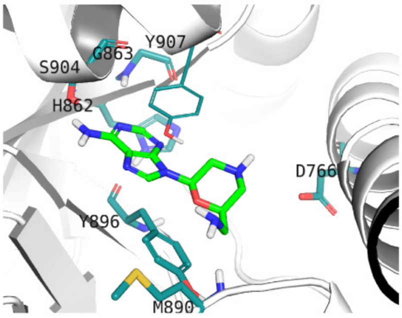

PARP1/H_2N-Mor-G

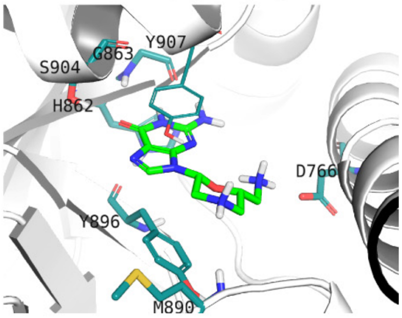

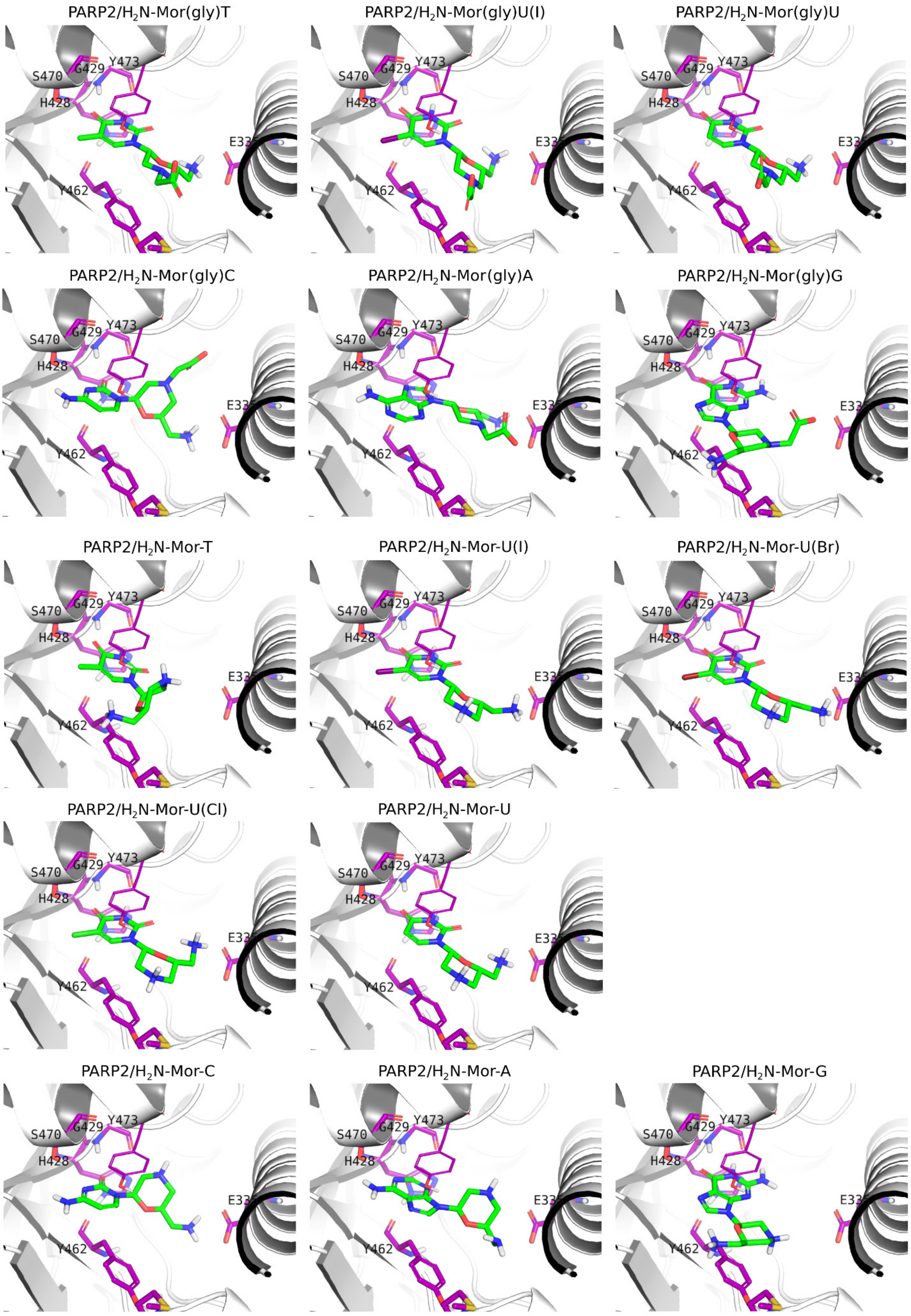
PARP2/H_2N-Mor(gly)T
PARP2/H_2N-Mor(gly)U(I)
PARP2/H_2N-Mor(gly)U
PARP2/H_2N-Mor(gly)C
PARP2/H_2N-Mor(gly)A
PARP2/H_2N-Mor(gly)G
PARP2/H_2N-Mor-T
PARP2/H_2N-Mor-U(I)
PARP2/H_2N-Mor-U(Br)
PARP2/H_2N-Mor-U(Cl)
PARP2/H_2N-Mor-U
PARP2/H_2N-Mor-C
PARP2/H_2N-Mor-A
PARP2/H_2N-Mor-G
S470
G429
Y473
H428
Y462

B) Predicted binding modes of compounds using RoseTTAFold All-Atom approach:

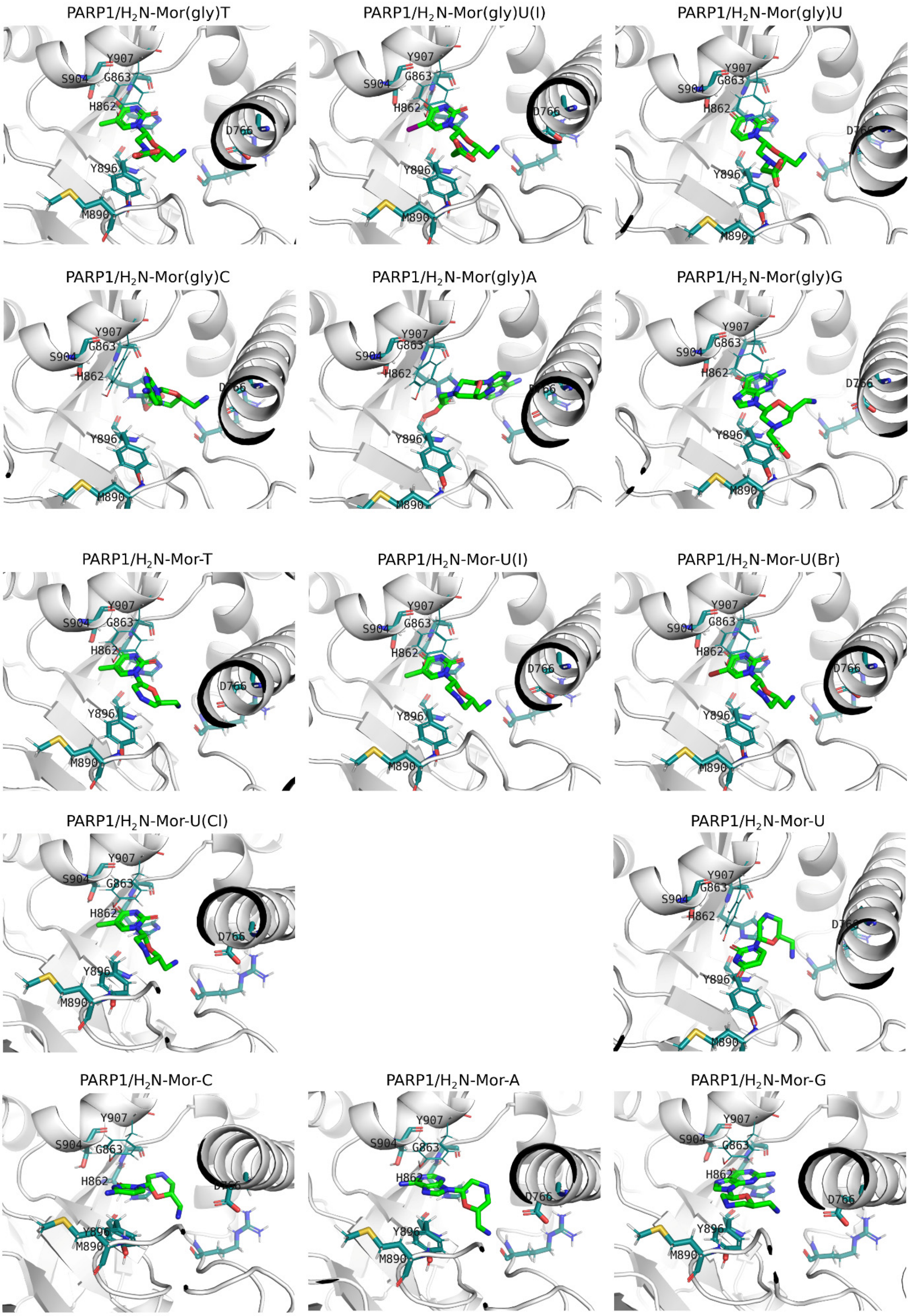

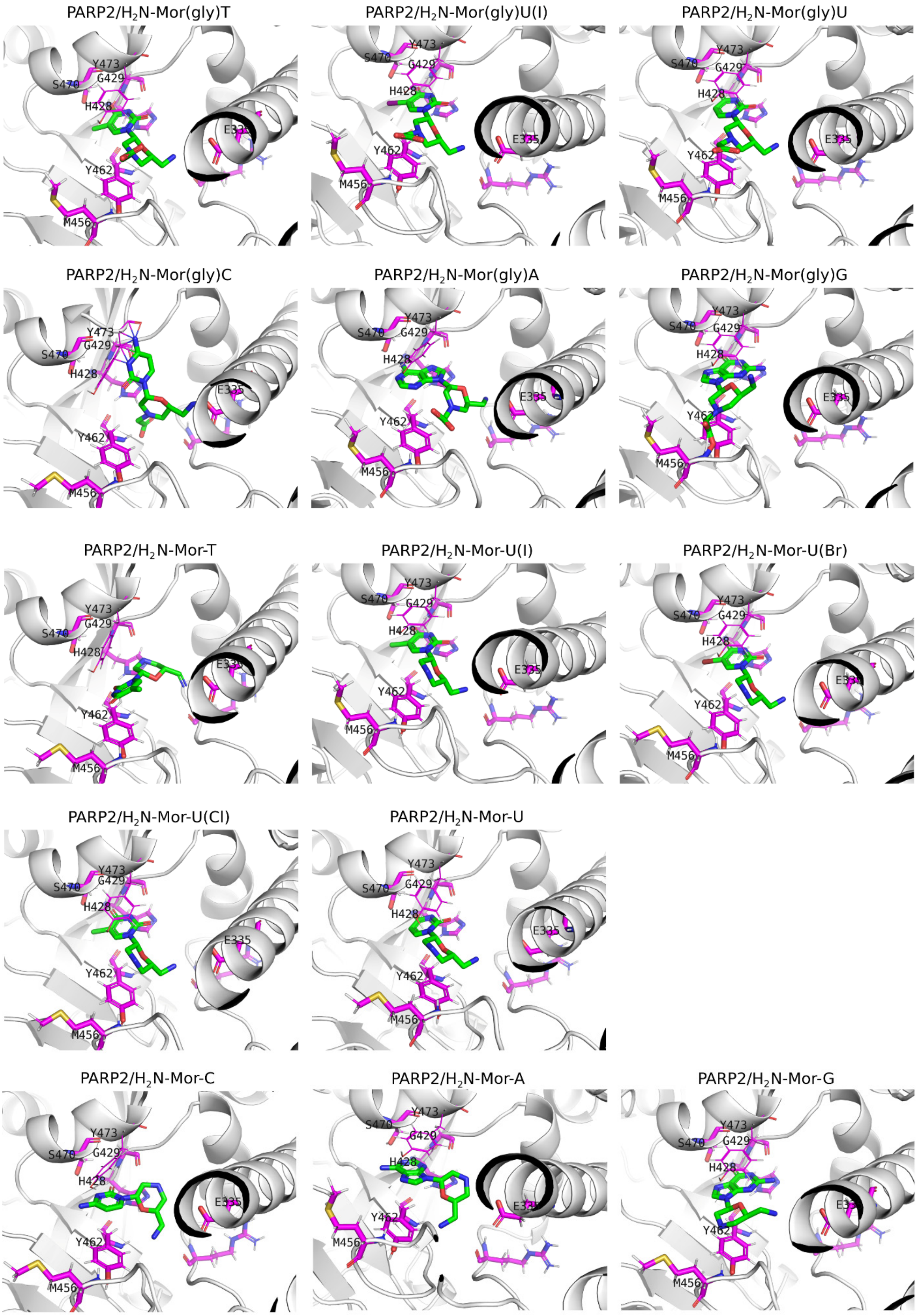

Figure S9. Binding modes of all compounds (shown in green) tested as inhibitors of PARP1 and PARP2 predicted by two different molecular docking approaches.

Table S1. The inhibition constants of the most active compounds against PARP1/PARP2 complex with NAD^+ substrate

#	Compound	$K_i(2)$*, μM	Enzyme
**8**	H_2N-Mor-T	40 ± 8	PARP1
**12**	H_2N-Mor-U(Cl)	300 ± 60	PARP1
**13**	H_2N-Mor-U(Br)	130 ± 30	PARP1
**14**	H_2N-Mor-U(I)	50 ± 10	PARP1
**14**	H_2N-Mor-U(I)	400 ± 70	PARP2

*All tested compounds appeared to be the mixed-type inhibitors. The $K_i(2)$ value (calculated as described in Figure S3 legend) reflects affinity for the enzyme complex with substrate.

Table S2. The combination index (CI) values for different concentrations of H_2O_2 and PARP inhibitors.

CI Data for Non-Constant Combo: H_2O_2+100/1000 µM **H_2N-Mor-U(I)**

Dose H_2O_2	Dose H_2N-Mor-U(I)	Effect	CI	Dose H2N-Mor-U(I)	Effect	CI
30.0	100.0	0.46	0.63310	1000.0	0.3	0.30026
50.0	100.0	0.47	0.57890	1000.0	0.32	0.24517
90.0	100.0	0.6	0.17778	1000.0	0.5	0.04618
150.0	100.0	0.68	0.08158	1000.0	0.61	0.01767
250.0	100.0	0.82	0.01487	1000.0	0.84	0.00135

CI Data for Non-Constant Combo: H_2O_2+100/1000 µM **H_2N-Mor-T**

Dose H_2O_2	Dose H_2N-Mor-T	Effect	CI	Dose H2N-Mor-T	Effect	CI
30.0	100.0	0.15	2.59576	1000.0	0.46	0.62541
50.0	100.0	0.21	1.70694	1000.0	0.59	0.26339
90.0	100.0	0.48	0.37499	1000.0	0.68	0.17241
150.0	100.0	0.61	0.27161	1000.0	0.79	0.09406
250.0	100.0	0.94	0.01519	1000.0	0.92	0.02575

CI Data for Non-Constant Combo: H_2O_2+30/50 µM **Olaparib**

Dose H_2O_2	Dose Olaparib	Effect	CI	Dose Olaparib	Effect	CI
30.0	30.0	0.32	1.13686	50.0	0.42	0.92889
50.0	30.0	0.51	0.42088	50.0	0.5	0.64452
90.0	30.0	0.6	0.31520	50.0	0.7	0.22661
150.0	30.0	0.85	0.06146	50.0	0.9	0.04027
250.0	30.0	0.96	0.01078	50.0	0.98	0.00464

CI Data for Non-Constant Combo: H_2O_2+100/800 µM **H_2N-Mor-C**

Dose H_2O_2	Dose H_2N-Mor-C	Effect	CI	Dose H_2N-Mor-C	Effect	CI
30.0	100.0	0.29	NaN	800.0	0.23	NaN
50.0	100.0	0.32	NaN	800.0	0.26	NaN
90.0	100.0	0.45	NaN	800.0	0.51	NaN
150.0	100.0	0.66	NaN	800.0	0.64	NaN
250.0	100.0	0.78	NaN	800.0	0.87	NaN

Table S3. Molecular docking scoring function prediction values

Compound	PARP1				PARP2			
	SP score[a]		XP score[a]		SP score[a]		XP score[a]	
	SP[b]	emodel[b]	XP[b]	emodel[b]	SP[b]	emodel[b]	XP[b]	emodel[b]
H_2N-Mor(gly)T	-11.1	-87.4	-10.9	-74.9	-10.0	-82.3	-10.2	-64.1
H_2N-Mor(gly)U(I)	-10.3	-85.6	-10.7	-75.5	-9.4	-82.0	-9.8	-70.1
H_2N-Mor-(gly)U(Cl)	-10.9	-83.8	-10.6	-69.7	-9.8	-77.3	-9.5	-63.6
H_2N-Mor-(gly)U(Br)	-10.7	-85.1	-10.9	-74.4	-9.6	-80.3	-11.1	-63.3
H_2N-Mor(gly)U	-10.5	-78.9	-10.3	-65.4	-9.4	-73.8	-9.1	-58.1
H_2N-Mor-(gly)A	-7.6	-68.2	-6.1	-63.5	-7.8	-74.3	-7.7	-60.3
H_2N-Mor-(gly)G	-10.2	-89.1	-8.2	-76.6	-9.4	-82.3	-10.6	-67.1
H_2N-Mor-(gly)C	-7.8	-67.0	-6.4	-54.1	-8.2	-66.6	-8.7	-54.1
H_2N-Mor-T	-8.6	-77.6	-8.2	-49.8	-9.3	-68.2	-8.6	-50.1
H_2N-Mor-U(I)	-8.2	-81.4	-7.4	-68.9	-8.7	-74.4	-9.0	-62.9
H_2N-Mor-U(Cl)	-9.3	-77.5	-9.1	-64.0	-9.4	-69.9	-9.2	-57.8
H_2N-Mor-U(Br)	-9.3	-79.4	-9.8	-63.1	-8.7	-68.4	-10.1	-58.0
H_2N-Mor-U	-9.1	-74.3	-9.8	-54.1	-8.6	-63.5	-8.5	-54.0
H_2N-Mor-A	-7.5	-63.4	-5.5	-54.2	-7.3	-54.9	-5.9	-54.6
H_2N-Mor-G	-8.9	-82.5	-7.4	-66.7	-9.0	-75.9	-7.9	-59.3
H_2N-Mor-C	-7.2	-66.5	-6.1	-46.1	-7.1	-56.5	-5.6	-47.8

[a]Scoring function
[b]Score

Table S4. Predicted alignment error (PAE) scores calculated by RoseTTAFold All-Atom approach

Compound	PARP1	PARP2
H_2N-Mor(gly)T	6.16	6.54
H_2N-Mor(gly)U(I)	6.49	7.02
H_2N-Mor-(gly)U(Cl)	7.35	8.76
H_2N-Mor-(gly)U(Br)	12.17	11.27
H_2N-Mor(gly)U	7.69	8.37
H_2N-Mor-(gly)A	13.09	14.93
H_2N-Mor-(gly)G	5.12	7.43
H_2N-Mor-(gly)C	4.56	5.49
H_2N-Mor-T	4.69	5.4
H_2N-Mor-U(I)	4.63	5.36
H_2N-Mor-U(Cl)	7.93	7.63
H_2N-Mor-U(Br)	6.49	7.03
H_2N-Mor-U	5.56	6.6
H_2N-Mor-A	6.75	7.77
H_2N-Mor-G	6.16	6.54
H_2N-Mor-C	6.49	7.02
